# Indole-Containing Natural Products 2019–2022: Isolations, Reappraisals, Syntheses, and Biological Activities

**DOI:** 10.3390/molecules27217586

**Published:** 2022-11-05

**Authors:** Syed Muhammad Umer, Mehwish Solangi, Khalid Mohammed Khan, Rahman Shah Zaib Saleem

**Affiliations:** 1 Department of Chemistry and Chemical Engineering, SBASSE, Lahore University of Management Sciences, Sector-U, DHA, Lahore 54792, Pakistan; 2 H. E. J. Research Institute of Chemistry, International Center for Chemical and Biological Sciences, University of Karachi, Karachi 75270, Pakistan; 3 Department of Clinical Pharmacy, Institute for Research and Medical Consultations (IRMC), Imam Abdulrahman Bin Faisal University, P.O. Box 31441, Dammam 31441, Saudi Arabia

**Keywords:** indole, natural products, cytotoxic, anticancer, antimicrobial, antiviral, other bioactivities

## Abstract

Indole alkaloids represent a large subset of natural products, with more than 4100 known compounds. The majority of these alkaloids are biologically active, with some exhibiting excellent antitumor, antibacterial, antiviral, antifungal, and antiplasmodial activities. Consequently, the natural products of this class have attracted considerable attention as potential leads for novel therapeutics and are routinely isolated, characterized, and profiled to gauge their biological potential. However, data on indole alkaloids, their various structures, and bioactivities are complex due to their diverse sources, such as plants, fungi, bacteria, sponges, tunicates, and bryozoans; thus, isolation methods produce an incredible trove of information. The situation is exacerbated when synthetic derivatives, as well as their structures, bioactivities, and synthetic schemes, are considered. Thus, to make such data comprehensive and inform researchers about the current field’s state, this review summarizes recent reports on novel indole alkaloids. It deals with the isolation and characterization of 250 novel indole alkaloids, a reappraisal of previously reported compounds, and total syntheses of indole alkaloids. In addition, several syntheses and semi-syntheses of indole-containing derivatives and their bioactivities are reported between January 2019 and July 2022.

## 1. Introduction

Despite the decreasing interest of modern pharmaceuticals in pursuing natural products as leads for new medicine [1], natural products and their derivatives still represent a significant fraction of approved drugs, with more than 22 compounds in the WHO list of essential medicine being sourced exclusively from flowering plants [2]. Furthermore, approximately 40% of all available medicine is either a natural product or a semi-synthetic derivative [3,4]. This ubiquity of natural products in medicine can be explained by their inherent nature as secondary metabolites. These compounds have been fine-tuned over millennia, acquiring biological functions to boost their host’s survivability [5]. Thus, these compounds can regulate endogenous defense mechanisms and interactions with other organisms, explaining their potency as antiviral, antibacterial, and antitumor agents [6]. Natural products also represent desired targets in synthesis due to their structural complexity or potent bioactivity.

Additionally, the pursuit of synthesizing natural products has led to novel reaction methodologies, catalyst design, and natural product-inspired synthetic analogs [7,8]. However, so far, only 5–15% of terrestrial plant species have been investigated as potential sources of therapeutic agents. Similarly, despite accounting for 90% of all-natural diversity, less than 1% of the microbial domain has been explored [1]. This minimal investigation of natural products’ sources (plants, insects, and microorganisms) represents a significant opportunity for chemists to scrutinize these sources and further discover nature’s offerings [9].

Due to their immense structural diversity, natural products are generally grouped into four broad categories: phenolics (phenylpropanoids), polyketides, terpenoids, and alkaloids [10,11]. Alkaloids are nitrogen-containing secondary metabolites, including important therapeutic agents such as hyoscyamine, quinine, or emetine [12,13]. Additionally, alkaloids are further divided into classes based on which nitrogen-containing moiety they possess; tropane, isoquinoline, imidazole, piperidine, and pyrrolizidine alkaloids are some examples [14].

Alkaloids containing indoles—indole alkaloids—are among the most significant alkaloid subsets, with more than 4100 different compounds [15]. Commercially available drugs having the indole moiety are ajmaline (antiarrhythmic agent), physostigmine (used to treat anticholinergic poisoning), and vincristine (antitumor agent) [16,17,18]. Similarly, many controlled substances such as ibogaine, psilocybin, and LSD (all powerful psychedelics) feature an indole moiety, demonstrating the group’s ubiquity in bioactive molecules [19].

This review aims to describe and summarize recent discoveries in indole-containing natural products and hopes to aid researchers by providing all relevant information (isolation method, synthesis, and bioactivity) in one place. For data collection, PubMed was used as the primary database (using “indole” and “natural product” as the key search words). Reports containing natural products with the indole or indoline rings were selected from about the last three and a half years (January 2019 to July 2022), while alkaloids containing structurally similar motifs such as sidelines, hydroindoles, and quinolines are not included. These articles described the isolations of novel indole-containing natural products, reappraisals of the structures of previously reported natural products, the total and semi-synthesis of indole alkaloids, and the syntheses of indole-containing derivatives. Bioactivities of all novel compounds and structure–activity relationship analyses (wherever available) are also included.

The articles reported 354 indole alkaloids, 250 isolated from 24 families, across the groups of living organisms. These alkaloids displayed immense biological activities, with cytotoxicity being the most common, followed by antibacterial, antifungal, antiplasmodial, and antiviral activities. Other bioactivities, including the promotion of glucose uptake, immunomodulatory effects (influencing the behavior of B-cells and DCs), analgesic effects, HDAC inhibition, and RANKL-induced multinuclear osteoclasts inhibition, were also reported. This variety in biological activities is matched by the diversity of structural classes present, with novel indole alkaloids possessing alstonine, aspidosperma, chetomin, corynanthean, epicoccin, eudistomin, fumiquinazoline, ibogaine, izumiphenazine A, macroline, notoamide, okaramine, paxilline, pleiocarpamine, sarpagine, serpentine, ursane, vincamine, and vobasine types of structures. Simultaneously, the article presents unique motifs and new classes, such as the psammocindoles.

## 2. Isolation of Novel Indole Alkaloids

### 2.1. Source Analysis

**Figure 1:**
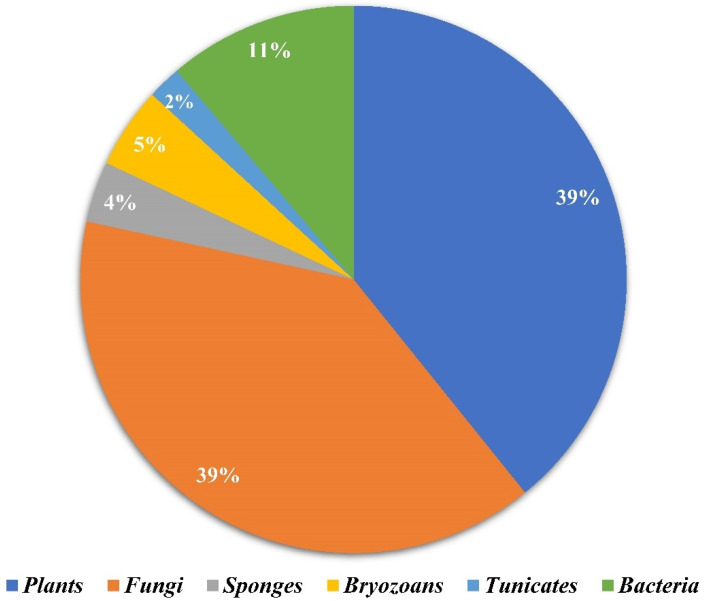
Distribution of the sources of novel indole alkaloids.

**Figure 2:**
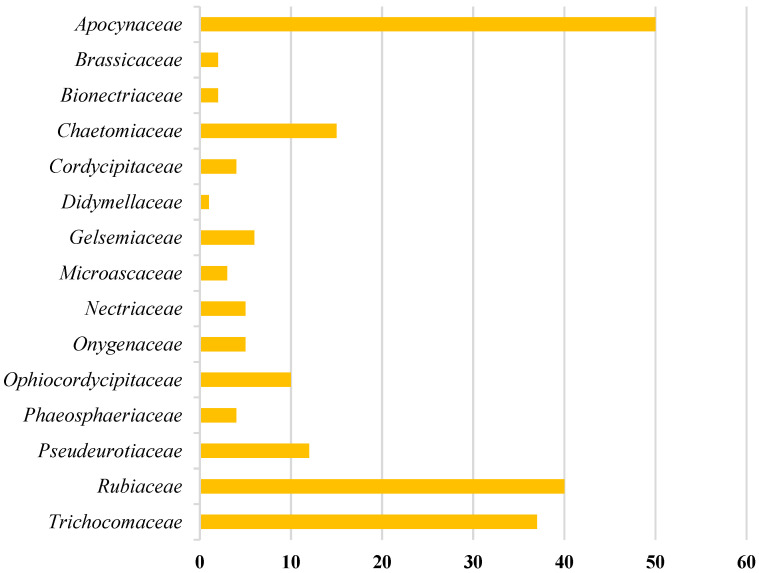
Distribution of indole alkaloids across plant, tree, and fungal sources.

Amongst 250, 98 of the compounds isolated (representing 39% of the total, Figure 1) were sourced exclusively from plants and trees (Apocynaceae, Rubiaceae, Gelsemiaceae, and Brassicaceae families; Figure 2). Similarly, 98 indole alkaloids were isolated from various fungi, with 37 of them being sourced from the Trichocomaceae family alone. Other fungal families such as Chaetomiaceae, Pseudeurotiaceae, and Ophiocordycipitaceae were sources of 15, 12, and 10 novel indole alkaloids, respectively (Figure 2). On the other hand, only 28 alkaloids were isolated from those in the Pseudomonadaceae and Streptomycetaceae families, producing 13 and nine, respectively. Other sources include bryozoans (Flustridae and Vesiculariidae), marine sponges (Coelosphaeridae, Irciniidae, and Petrosiidae), and tunicates (Polyclinidae, Polycitoridae, Didemnidae, and Pseudodistoma) from which 12, nine, and five indole alkaloids, respectively, were isolated (Figure 1).

### 2.2. Isolation Methods

The isolation methods generally included using organic solvents (dichloromethane, ethanol, ethyl acetate, or methanol) to extract a crude mixture of alkaloids directly from the source (bacteria, fungi, dried plant material, etc.). This mixture was then concentrated and resuspended in acidic media (acidified using HCl, H_2_SO_4_, or tartaric acid). Next, the organic layer containing fats and other dissolved material was removed, while the aqueous layer was isolated and basified using NH_3_, NH_4_OH, or NaOH. Subsequently, the organic layer was concentrated and purified by column chromatography, affording pure alkaloids.

### 2.3. Structural Novelties

Of the 250 novel indole alkaloids isolated and analyzed (Table 1), 207 of them were monoindole alkaloids (MIAs), 41 were bisindoles, and only two were trisindoles (238 and 239). Similarly, 40 compounds were monoterpenoids, and 19 were diterpenoids. Some of the alkaloids discovered had structures like or derived from alstonine, aspidosperma, chetomin, corynanthean, epicoccin, eudistomin, fumiquinazoline, ibogaine, izumiphenazine A, macroline, notoamide, okaramine, paxillin, pleiocarpamine, sarpagine, serpentine, ursane, vincamine, and vobasine. In addition, novel compounds such as a long polypeptide bearing a C-terminus tryptamine (**63**), two oxindole alkaloid glycosides (**92** and **93**), an oxazolidinium-containing indole found in nature (**177**), and a methylenedioxy dibromoindole alkaloid with antiosteoporosis activity (**233**) were isolated.

**Figure 3:**
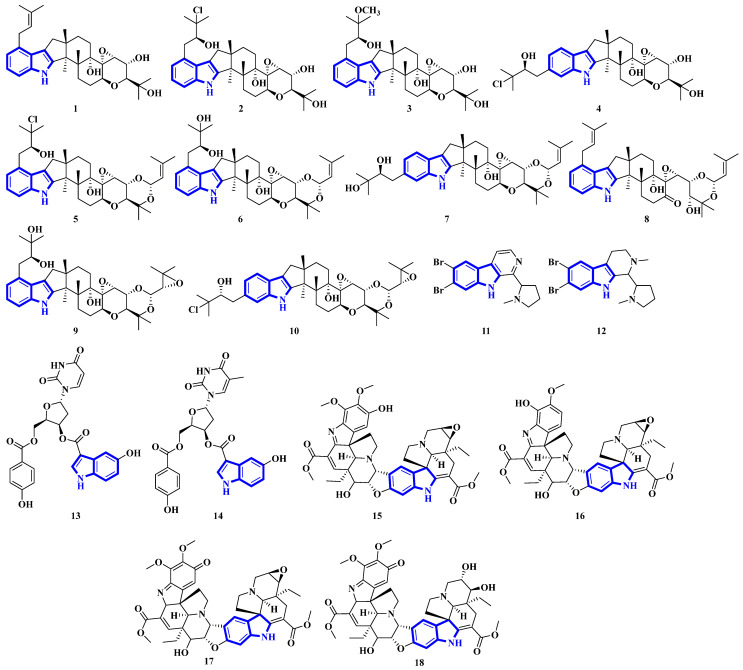
Novel indole alkaloids **1** to **18**.

Compounds **1** to **10** are prenylated indole diterpenoids, with chlorinated analogs (**2**, **4**, **5**, and **10**) being the first indole diterpenes isolated from fungi. These alkaloids resemble terpendole I and terpendole C, themselves biosynthesized from an alkaloid, paspaline [20]. Compounds **11** and **12** are eudistomin-type alkaloids isolated from the tunicate families Eudistoma and Pseudodistoma, whereas compounds **13** and **14** are novel nucleosides isolated from Didemnidae, also a tunicate [21]. Lastly, compounds **15** and **16** are bisindole alkaloids, while compounds **17** and **18** are MIAs, with all four being isolated from the leaves of *Tabernaemontana corymbosa* (Figure 3) [22].

**Figure 4:**
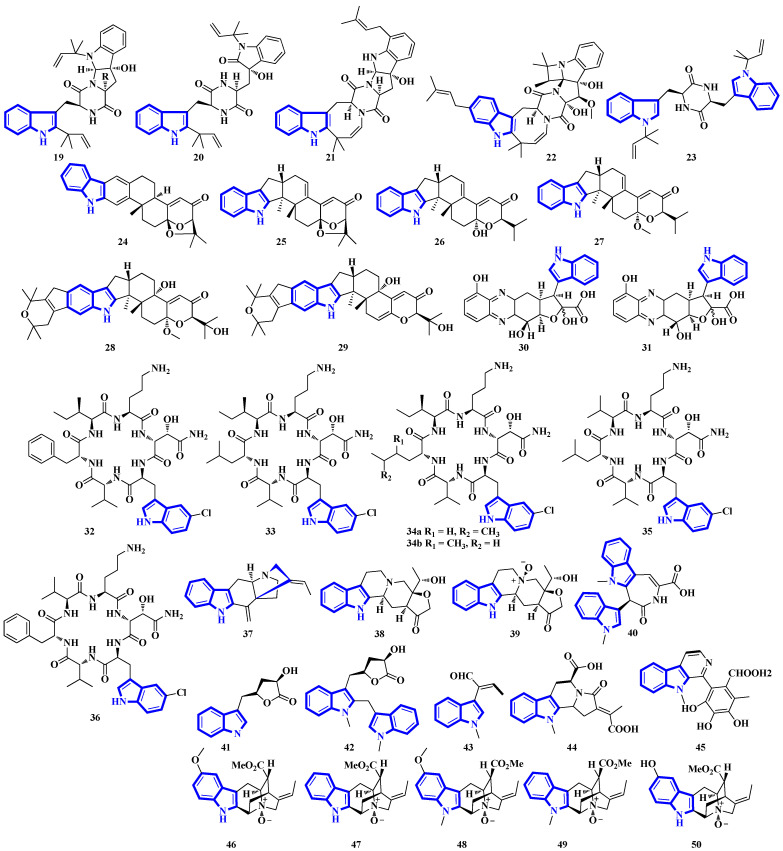
Novel indole alkaloids **19** to **50**.

Compounds **19** to **23** were isolated from *Aphanoascus fulvescens*, obtained from goose dung, and represent okaramine-type bisindoles (Figure 4) [23]. Compounds **24** to **29** are indole diterpenoids in which compound **24** is a new scaffold with a rare carbazole unit forming a 6/5/6/6/6/6/5 heterocyclic system bearing an aromatic ring C [24]. Compounds **30** and **31** are izumiphenazine A-type alkaloids containing a novel 5-hydroxyquinoxaline and alpha-keto acid motif [25]; however, compounds **32** to **36** are chlorinated cyclic hexapeptides. Unfortunately, the structure of compound **34** could not be completely resolved since minute amounts of it were isolated (**34a** and **34b**) [26]. Compounds **37** to **39** were isolated from the stem bark of *Kopsia arborea*. Compound **37** is an unusual pentacyclic monoterpenoid alkaloid with a new carbon–nitrogen skeleton. Furthermore, compounds **38** and **39** are pentacyclic corynanthean-type alkaloids that contain a hydroxyethyl-substituted tetrahydrofuranone ring [27]. Compounds **40** and **42** are bisindoles, with the former having a β-carboline motif. Compounds **44** and **45** are demethylated derivatives of chaetogline A and F (demethylation on 19-O and 20-O, respectively) [28]. Compounds **46** to **50** were isolated using the Computer-Assisted Natural Products Anticipation (CANPA) workflow. It is the first nonpeptidic molecular networking-based natural product chemistry workflow. It generated the targeted structures of the alkaloids even before their isolations. Additionally, compounds **46** to **50** are sarpagine-type *N*-oxide alkaloids [29].

**Figure 5:**
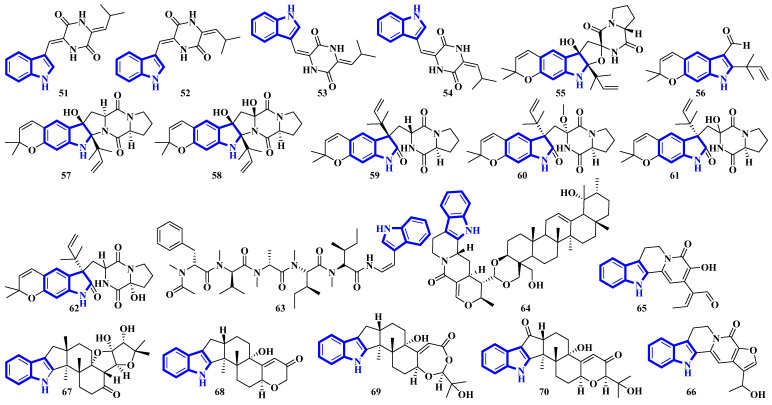
Novel indole alkaloids **51** to **70**.

Compounds **51** to **54** are unsaturated diketopiperazine derivatives [30], and **55** to **62** are linearly fused prenylated indole alkaloids (Figure 5). Compound **55** is the first diketopiperazine derived from d-proline and l-tryptophan and possesses an unprecedented C-11-spiro-fused 6/6/5/5/6/5 ring system. Similarly, compound **56** is the first linearly fused 6/6/5 tricyclic prenylated indole alkaloid discovered [31]. Compound **63** is a highly modified linear hexapeptide with an unusual per methylation of the amino-acid backbone. It is the most extended polypeptide with a tryptamine C-terminus and displays acetylation on its N-terminus, a rare feature among natural and fungal products [32]. Alkaloids **64** to **66** were isolated from the heartwood of *Nauclea latifolia*. Compound **64** contains an unusual monoterpene indole alkaloid unit condensed with an ursane-type pentacyclic triterpenoid motif that is further bonded to a six-membered-ring acetal; furthermore, monoterpenoid **66** is a constitutional isomer of compound **65** [33]. Compounds **67** to **70** were isolated from a *Penicillium* fungus extracted from *Meretrix lusoria*, a bivalve mollusk (Figure 5). Compound **67** is an indolediterpenoid with a unique 6/5/5/6/6/5/5 heptacyclic ring system. Furthermore, compounds **68** and **69** are paxilline-type alkaloids with some modifications; compound **68** contains three fewer carbons (C-23/24/25), while compound **69** contains an additional oxygen atom between C-21 and C-22 than paxilline. Compound **69** also has an unusual 6/5/5/6/6/7 hexacyclic ring system with a 1,3-dioxepane ring, a rarity among natural products [34].

**Figure 6:**
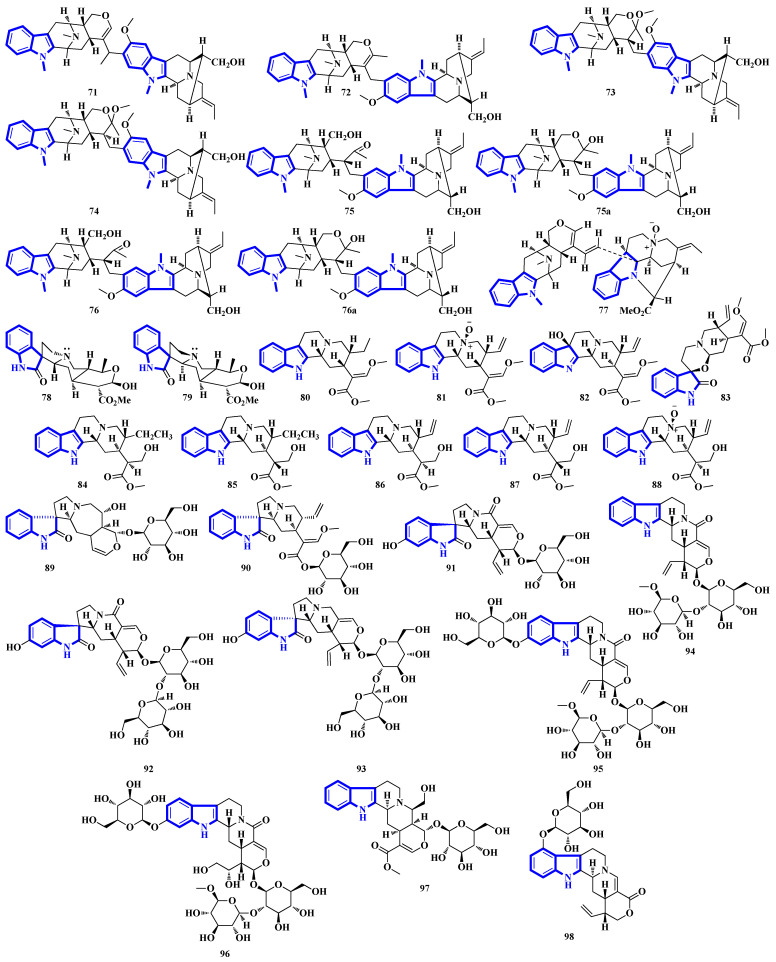
Novel indole alkaloids **71** to **98**.

Bisindoles **71** to **77** were isolated from the stem bark of *Alstonia penangiana* (Figure 6). Compound **77** is a macroline-pleiocarpamine-type alkaloid, and compound **74** is a constitutional isomer of compound **73**. Compounds **75** and **76** exist as an equilibrium mixture of their ring-opened and hemiketal (**75a** and **76a**) states. The hemiketal versions are more prevalent in their solid forms, while dissolving the alkaloids in CDCl_3_ yields ring-opened forms [35]. Compounds **78** and **79** were isolated from the leaves of *Uncaria longiflora*; nonetheless, compounds **80** to **88** and **89** to **98** were isolated from the hooks and hook-bearing stems of *Uncaria rhynchophylla*, respectively. Interestingly, all 21 alkaloids were monoterpenoids, with compounds **89** to **98** containing gluco-conjugated motifs [36,37]. Furthermore, compound **89** is a new subtype of oxindole alkaloids with a seven-membered D-ring; compound **98** contains a rare glucosyl moiety at C-9. Compounds **92** and **93** represent the first two oxindole alkaloid diglycosides reported [38].

**Figure 7:**
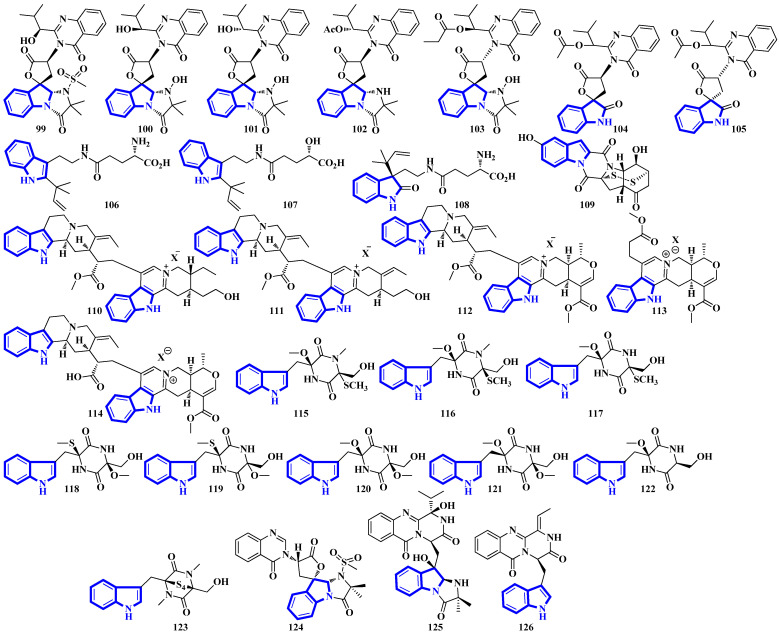
Novel indole alkaloids **99** to **126**.

Quinazoline-containing compounds **99** to **105** were isolated from a fungus in the Aspergillus genus extracted from *Sanguinolaria chinensis*, a bivalve mollusk. Additionally, compounds **99** to **103** all contain a 5/5/6/5/6/6 hexacyclic ring system and 1-hydroxy-2-methyl propyl groups at C-26. Some have an acylated hydroxy, while compound **99** possesses a rare amino sulfonyl group. Removing the C-14/C-15/N-16 fragment, and the two methyl groups bonded to C-15 in compounds **99** to **103** yields the structural framework for compounds **104** and **105** [39]. Compounds **106** to **108** are water-soluble glutamic acid derivatives [40]. Compound **109** was isolated from *Epicoccum nigrum*, extracted from the gills of *Amphilophus*. It contains an aromatic indole motif, a rarity among the epicoccin type of epipolythiodioxopiperazines (ETPs) [41]. Alkaloids **110** to **114** were isolated from the aerial parts of *Picralima nitida*. Compounds **110** to **113** are serpentine-type bisindoles, while compound **114** is analstonine-type MIA [42]. Similarly, compounds **115** to **123** are also ETPs. These were isolated from *Chaetomium cochliodes*, which were extracted from animal manure. Compounds **115** to **123** are chetomin-type MIAs, with compound **123** possessing a unique tetra-sulfur bridge [43]. Compounds **124** to **126** are fumiquinazoline-type alkaloids isolated from *Scedosporium apiospermum*, extracted from the inner tissue of *Lobophytum crissum*, a coral (Figure 7) [44].

**Figure 8:**
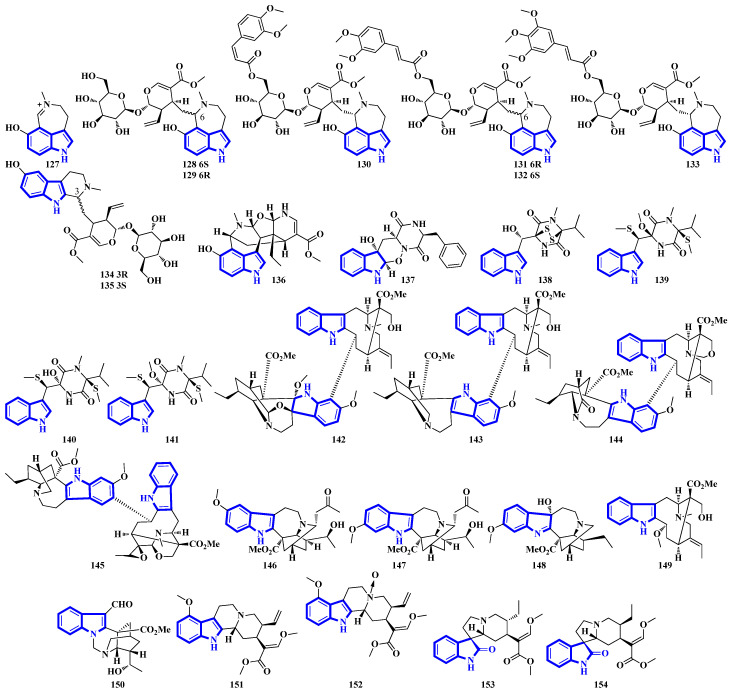
Novel indole alkaloids **127** to **154**.

Alkaloids **127** to **136** were isolated from the leaves of *Psychotria nemorosa* (Figure 8). Compounds **127** to **133** and **136** are azepine-type indole alkaloids, while compounds **134** and **135** are β-carboline derivatives [45]. Compound **137** is an indole diketopiperazine alkaloid with an unusual pyrazino [1′,2′:2,3][1,2]oxazino [6,5-b]indole tetraheterocyclic ring system [46]. Compounds **138** to **141** are thiodiketopiperazine alkaloids isolated from *Phaeosphaeria fuckelii*, extracted from *Phlomis umbrosa*. They all contain an unusual β-(oxy)thiotryptophan motif [47]. Compounds **142** to **150** were isolated from the twigs and leaves of *Tabernaemontana corymbose*. Compounds 142 to 145 are vobasinyl-ibogaine-type alkaloids, with compound **142** additionally containing an unusual 1,3-oxazinane motif [48]. Compounds **151** and **152** were isolated from *Mitragyna speciosa*, while compounds **153** and **154** were isolated from Havil. Rubiaceae. Compound **151** is a diastereomer of paynantheine, while compound **152** is a paynantheine-type N-4 oxide. Furthermore, compounds **153** and **154** are spirocyclic oxindoles, and are C-3 epimers of rhynchophylline and corynoxine B, respectively [49].

**Figure 9:**
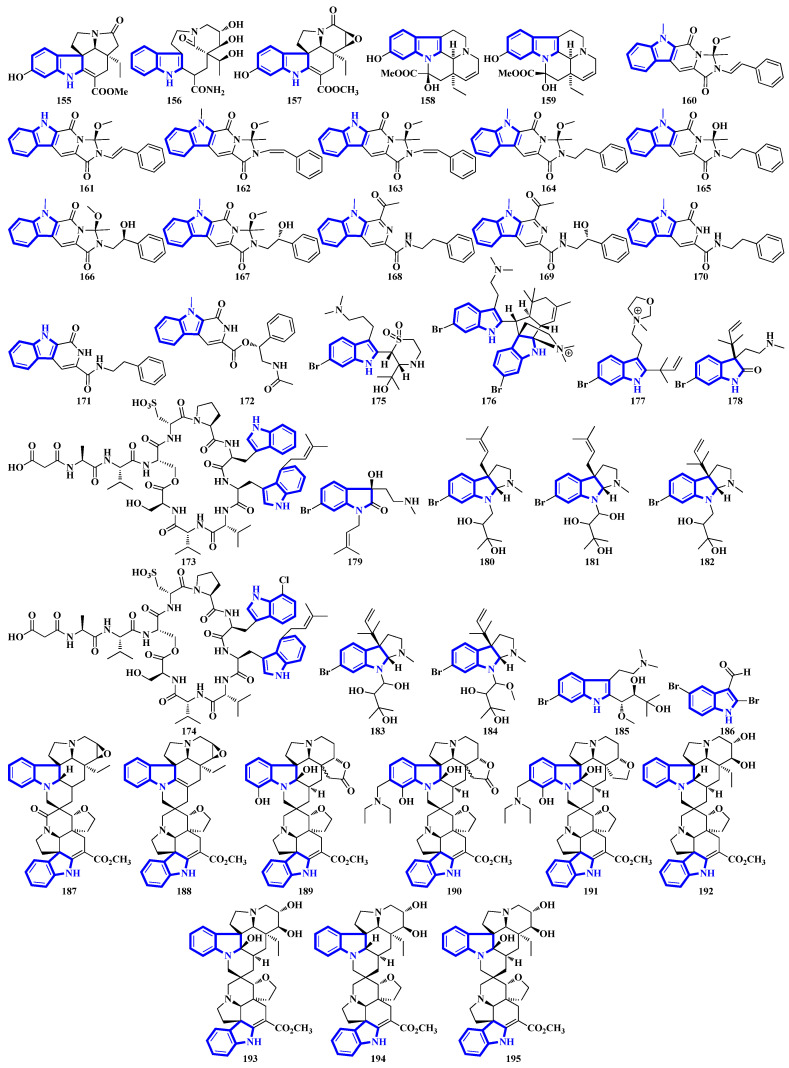
Novel indole alkaloids **155** to **195**.

Compounds **155** to **159** were isolated from the twigs and leaves of *Melodinus hemsleyanus* (Figure 9). Compound **155** is an unusual aspidosperma-type alkaloid possessing a 6/5/6/5/5 pentacyclic architecture with a smaller E ring (loss of CH_2_), while compounds **158** and **159** are vincamine-type alkaloids [50]. Compounds **160** to **172** are β-carboline alkaloids that were isolated from *Actinoalloteichus* that was obtained from sea mud. Compounds **160** to **167** share a novel indole–pyridone–imidazole tetracyclic framework. Additionally, compounds **164** to **167** are analogs of **160** but lack Δ15 and have different substitutions at C-13 and C-16 [51]. Compounds **173** and **174** are krisynomycins isolated from *Streptomyces canus* and obtained from sand in the Kalahari Desert. They are non-ribosomal cyclic depsipeptides bisindoles with prenylated and chlorinated tryptophan monomers [52]. Compounds **175** to **185** are bromotryptamine alkaloids, whereas **175** possesses a rare 1,4-thiazine-1,1-dioxide group. Furthermore, compound **177** is the first oxazolidinium-containing indole from nature [53]. Compound **186** was isolated from Amathialamourouxi, a bryozoan found in rock pools. It is a simpler molecule; its name is 2,5-dibromo-1-methyl-1*H*-indole-3-carbaldehyde [54]. Compounds **187** to **195** are monoterpenoid indole alkaloids that were extracted from the leaves of *Tabernaemontana pachysiphon*. They are all aspidosperma–aspidosperma-type alkaloids and possess a rare spiro heterocycle between their two constituent units. Compounds **190** and **191** contain an indole ring fused with an (*N*,*N*-diethyl)methyl amino group [55].

**Figure 10:**
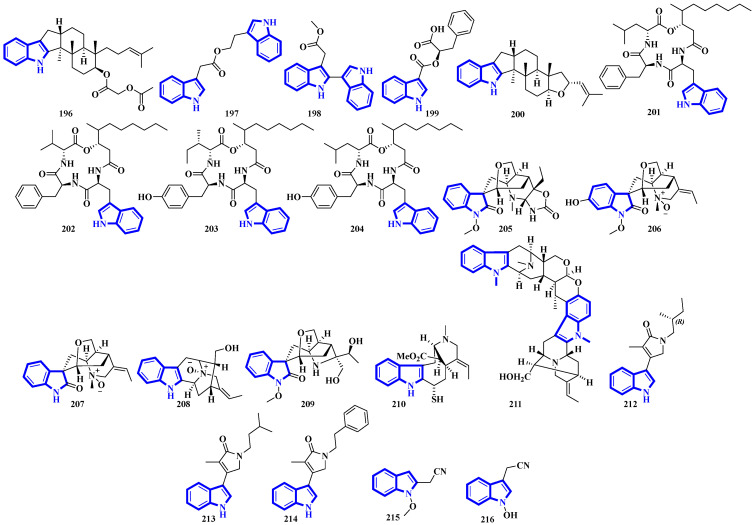
Novel indole alkaloids **196** to **216**.

Alkaloids **196** to **200** were isolated from *Fusarium* obtained from the inner tissue of *Acanthaster planci*, and compounds **197** and **198** are bisindoles [56]. Compounds **201** to **204** are cyclotetradepsipeptides isolated from *Beauveria* fungi and obtained from the fresh stems of *Gypsoplaca macrophylla*. These alkaloids contain a 3-hydroxy-4-methyldecanoic acid (HMDA) group [57]. Compounds **205** to **209** are monoterpenoid indole alkaloids that were isolated from the stems of *Gelsemium elegans*. Furthermore, compound **205** is a novel triamino alkaloid with a novel 6/5/7/6/6/5 hetero hexacyclic ring system bearing a unique hexahydrooxazolo [4,5-b]pyridin-2(3*H*)-one motif. In contrast, compound **208** is the first *N*-oxide of a sarpagine-type alkaloid isolated from a plant in the *Gelsemium genus* [58]. Compound **210** was isolated from the stems of *Mostuea brunonis* and is a novel sulfur-containing vobasine-type indole alkaloid. It is among the rare monomeric vobasines lacking oxygen at C-3 [59]. Compound **211** was isolated from the stem bark of *Alstonia penangiana* and is a linearly fused macroline-sarpagine-type bisindole [60]. Compounds **212** to **214** are indole-γ-lactams and represent psammocindoles, a new class of indole alkaloids derived from three amino acids (isoleucine, leucine, and phenylalanine) [61]. Compounds **215** and **216** were isolated from the roots of *Isatis indigotica*, and they contain an indole acetonitrile motif (Figure 10) [62].

**Figure 11:**
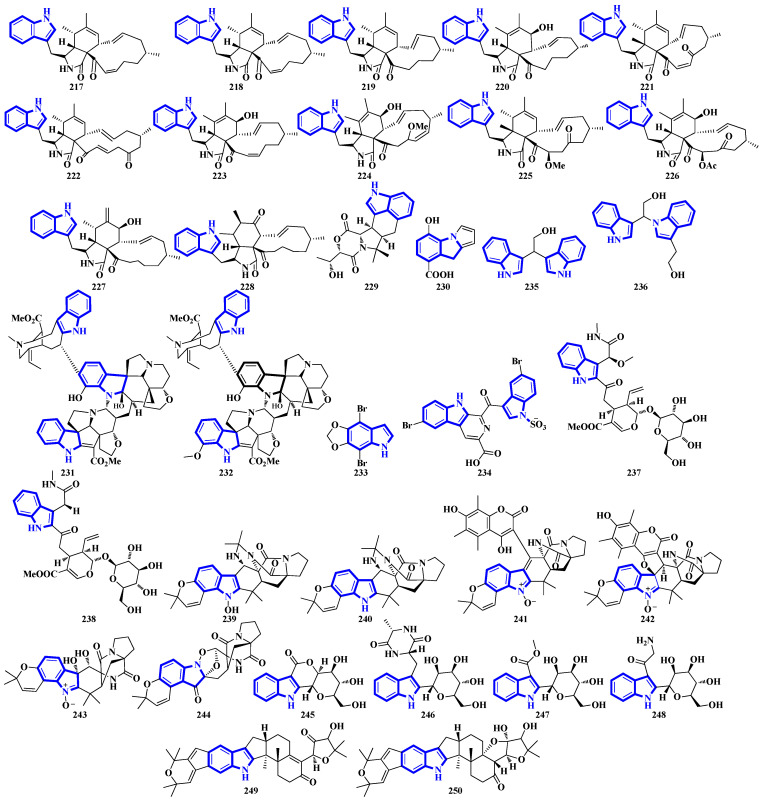
Novel indole alkaloids **217** to **250**.

Compounds **217** to **228** are novel [11]-chaetoglobosins isolated from *Pseudeurotium bakeri*, obtained from macrocoma moss (family: Orthotrichaceae). The alkaloids, containing an 11-membered macrocyclic ring, represent a rare motif as only seven naturally occurring [11]-chaetoglobosins have been reported [63]. Compound **229** is a novel indole-linked 2,5-diketomorpholine alkaloid with an unprecedented 6/5/6/6/5 core, while compound **230** contains an indole fused with pyrrole and possesses a 6/5/5 core [64]. Compounds **231** and **232** were isolated from the bark of *Voacanga africana*, and they are the first monoterpene trisindole alkaloids containing a vobasine–aspidosperma–aspidosperma skeleton [65]. Similarly, compound **233** is the first methylenedioxy dibromoindole alkaloid exhibiting antiosteoporosis activity [66]. Additionally, compound **234** is a dibrominated β-carboline sulfamate, one of the only 11 indole sulfamate-containing marine natural products reported in the Marin Lit. database [67]. Compounds **235** and **236** were products of the heterologous expression of the EDB gene cluster (found in *P. fluorescens* NZI7) in *E. coli*. The metabolites are isoindoles containing an ethanol group [68]. Compounds **237** and **238** were isolated by processing the whole *Ophiorrhiza japonica* plant representing two novel glycosides [69]. Compounds **239** to **244** are notoamide-type alkaloids isolated from *Aspergillus sclerotiorum*, extracted from a gorgonian coral. Compounds **239** and **240** possess a unique 2,2-diaminopropane group, while compounds **241** and **242** are novel notoamide hybrids having a coumarin moiety. Compound **244** is a new highly oxidized notoamide scaffold [70]. Compounds **245** to **248** are indole-C-mannopyranoside alkaloids: rare indole C-glycosides in which an α-D-mannose residue is connected to the indole ring at C-2 [71]. Compounds **249** and **250** are diterpenoid indole alkaloids isolated from *Penicillium oxalicum* obtained from the mantis shrimp, *Oratosquilla oratoria*. Compound **249** is the first indole-diterpenoid derivative with a 4-hydroxy-5,5-dimethyldihydrofuran-3-one as a side-chain (6/5/6/5/5/6/6-5 system). Compound **250** is a hemiketal containing a unique 6/5/6/5/5/6/6/5/5 ring system (Figure 11) [72].

### 2.4. Structure Reappraisals

Due to the stereochemical richness of natural products and structural complexity, extensive spectroscopic analyses are required to elucidate their complete structures. Occasionally, some fragments within the compounds are misidentified, possibly due to a lack of spectroscopic data resulting from insufficient quantities of the compound to analyze. Later, due to contradictory results (often emerging from the synthesis of homologs or the analysis of their decomposition products), the postulated structures of compounds are reevaluated, and the errors in the initially proposed structures are rectified.

**Figure 12:**
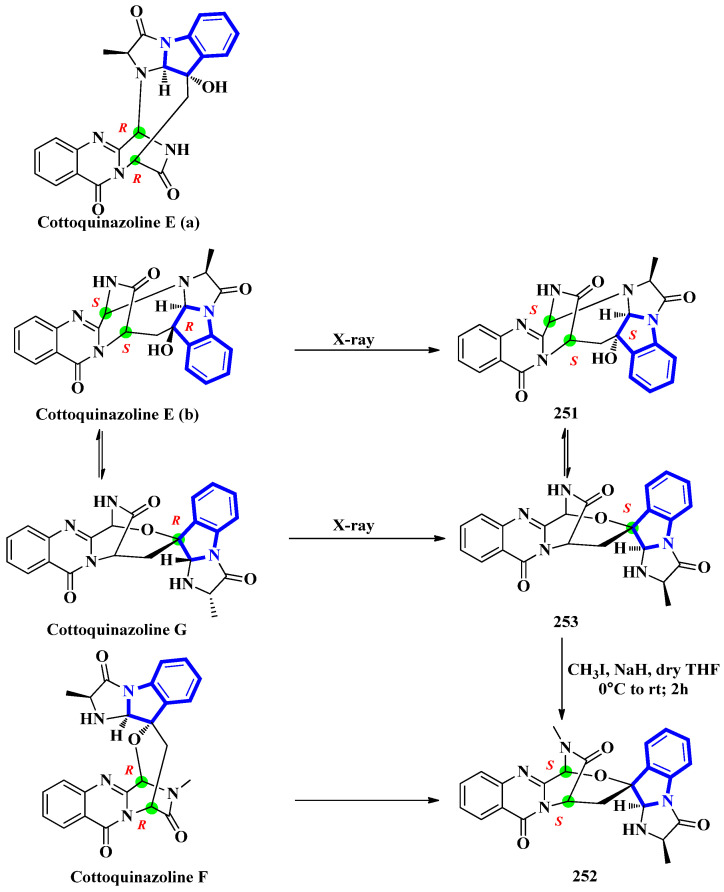
Reappraised structures of cottoquinazolines (E–G) **251–253**.

Cottoquinazolines E-G (**251** to **253**) were isolated from the fungus *Neosartorya fischeri*, found in the medicinal arthropod *Cryptotympana atrata* [73]. The fumiquinazoline-type alkaloids were reinvestigated due to unresolved absolute configuration of compounds **251** and **252**. Extensive spectroscopic analysis, electronic circular dichroism data, and X-ray crystallography confirmed the absolute configurations of all three indole alkaloids (Figure 12). Cottoquinazoline E had two posited steric conformations: cottoquinazoline E (a) with C-3R, C-14R, and C-16S configurations or cottoquinazoline E (b) with C-3S, C-14S, and C-16R configurations. In addition, spectroscopic studies confirmed the structure of **251** and its configurations (C-3S, C-14S, and C-16S). Similarly, the absolute configurations of **252** were changed from C-3R and C-14R to C-3S and C-14S, and **253** was modified from C-16R to C-16S [74].

**Figure 13:**
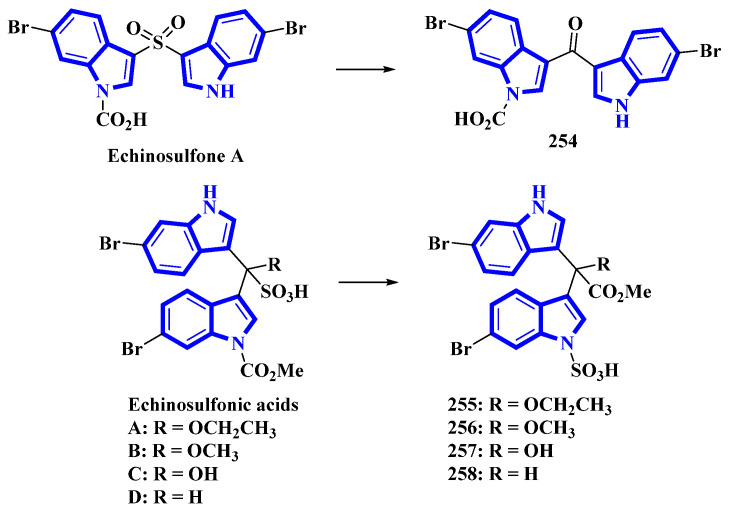
Reappraised structures of echinosulfone A and echinosulfonic acids (A–D) **254–258**.

Echinosulfone A (**254**) and echinosulfonic acids A–C (**255–257**) were initially isolated from *Echinodictyum*, a marine sponge [75]. Similarly, echinosulfonic acid D (**258**) was isolated from a *Psammoclema* sponge, and its structure was assigned on the basis of the previous report [76]. However, discrepancies arose when decomposition products of **254** were analyzed using X-ray diffraction. Thus, the original alkaloids were reisolated from sponges in the *Crella* genus. Their structures were re-evaluated using 1D and 2D NMR data in conjunction with MS fragmentation, X-ray crystallography, and DFT predictions of ^13^C-NMR shifts. For compound **254**, the original sulfonyl bridge between the two indole monomers was modified to a carbonyl bridge, while the N-1-bonded carboxylic acid was replaced with an N-1-bonded sulfonic acid (Figure 11). Similar changes were made in compounds **255** to **258**, where the C1ʹʹ-bonded sulfonic acid was changed with the C1ʹʹ-bonded methoxycarbonyl group. The N-1-bonded methoxycarbonyl was replaced with an N-1-bonded sulfonic acid moiety (Figure 13) [77].

**Figure 14:**
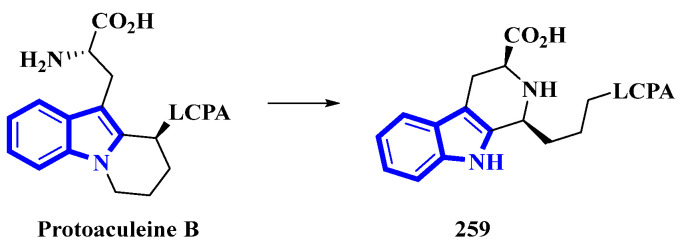
Reappraised structure of protoaculeine B **259**.

Protoaculeine B (**259**) is the N-terminal residue of aculeine B, a peptide toxin that is a potent membrane disruptor [78]. Aculeines are found in the aqueous extracts of *Axinyssa aculeata*, a marine sponge. Aculeine B consists of a 44 amino-acid long sequence (containing three disulfide bonds), connected to a long-chain polyamine (LCPA; consisting of a 1,3-propanediamine oligomer) by a tryptophan-derived amino acid (**259**). A homolog of **259** was synthesized, but its spectroscopic and chemical properties differed significantly from its natural counterpart. Thus, the structure of compound **259** was re-evaluated, and its C-3-bonded amino-acid chain was modified into a fused pyridine-2-carboxylic acid ring bonded to the LCPA via a propane group (Figure 14) [79].

**Figure 15:**
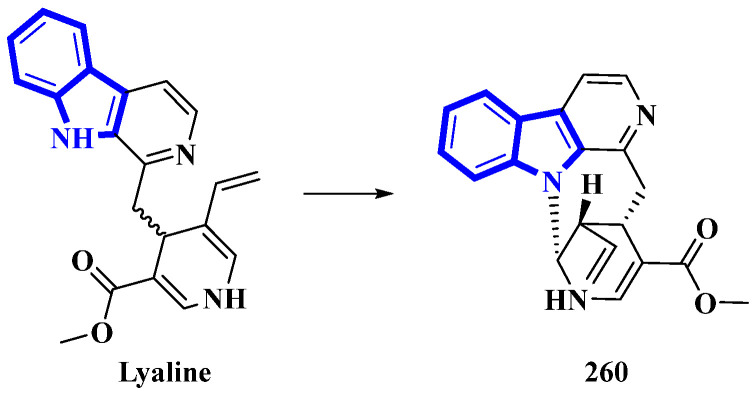
Revised structure of lyaline **260**.

Lyaline (**260**) was isolated from *Pauridiantha paucinervis*, and was reported as an unusual harman-1,4-dihydropyridine derivative [80]. However, efforts to synthesize the monoterpene indole alkaloid resulted in discrepancies (the synthetic product was extremely unstable; nevertheless, the natural analog did not show any degradation) [81]. Thus, the absolute configuration of C-15 was elucidated as S, and a single bond between C-21 and N-1 was added. As Δ20 from the original structure was reduced, it was revised as S (Figure 15). Lastly, considering the revised structure of **260**, it was established as the first naturally occurring nacycline analog [82].

## 3. Synthesis of Indole Alkaloids

### 3.1. Total Synthesis of Indole Alkaloids

**Figure 16:**
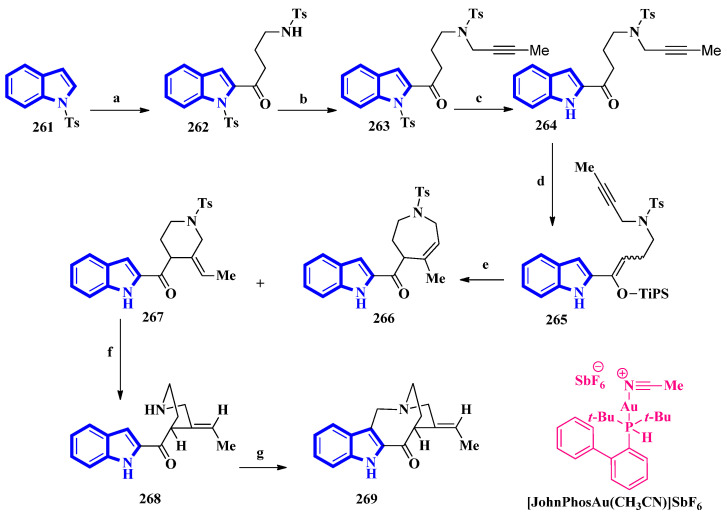
The total synthesis of (±)-conolidine **269**. (a) *n*-BuLi, THF, −78 °C, 5 min, then rt, 1 h, then *N*-tosylpyrrolidonein THF, −30 °C to −15 °C, 4 h, 48%; (b) K_2_CO_3_, 1-bromo-2-butyne, CH_3_CN, 80 °C, 94%; (c) TBAF, CH_3_CN 35 °C, 15 h, 93%; (d) TiPSOTf, 2,6-lutidine, 35 °C, 5 h, 97%, E:Z = 8:92; (e) [JohnPhosAu(CH_3_CN)]SbF6, H_2_O, toluene, 60 °C, 2 h, 15% for 266, 73% for 267; (f) Sodium naphthalenide, THF, −78 °C, 86%; (g) (CH_2_O)n, TFA, CH_3_CN, reflux, 2 h, 82%.

(±)-Conolidine (**269**), a potent nonopioid analgesic, was synthesized in six steps without any nonstrategic redox manipulations. It was isolated from the stem bark of *Tabernaemontana divaricate* in 2004 [83], and its first total synthesis was carried out in 2011 with a nine-step synthetic route in an 18% overall yield [84]. The latest total synthesis attempts primarily utilized gold(I)-catalyzed Conia-ene and Pictet–Spengler reactions, producing **269** in six steps in overall 19% yield (Figure 16). They also used DFT calculations to develop a successful scheme [85].

For the total synthesis of compound **269**, compound **261** was treated with *n*-butyllithium and dropwise addition to *N*-tosylpyrrolidone affording **262** that underwent a nucleophilic reaction with 1-bromo-2-butyne to give compound **263**. Next, compound **263** was treated with TBAF to remove the *N*-1-tosyl group producing compound **264**. Afterward, **264** was added to TiPSOTf in 2,6-lutidine, affording a pair of stereoisomers of **265** (E:Z = 8:92). Its gold-catalyzed reaction using [JohnPhosAu(CH_3_CN)]SbF6 produced compounds **266** and **267**. The latter was exposed to sodium naphthalenide to deprotect its N-2 tosyl group affording **268**. Lastly, a Pictet–Spengler reaction using TFA and paraformaldehyde in acetonitrile was used to convert **268** into **269** [85].

**Figure 17:**
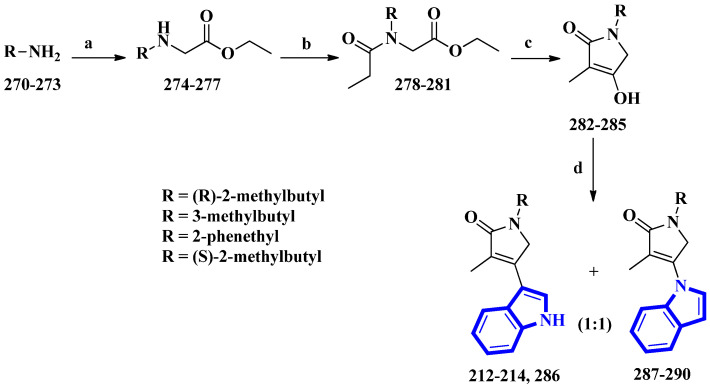
The total synthesis of psammocindoles (A–D) **212–214**, **286**, and their isomers **287–290**. (a) Ethyl bromoacetate, DCM, Et_3_N, rt, 2 h, 90–93%; (b) Propionyl chloride, Et_3_N, 0 °C to rt, 1.5 h, 94–95%; (c) NaH, THF, reflux, 12 h, 83–86%; (d) Indole, BF_3_-Et_2_O, 4 Å MS, PhCl, 100 °C, 1.5 h, 27–31%.

Psammocindoles A and B (**212** and **213**) significantly increased adiponectin production. Thus, to further investigate their pharmacology and assign the absolute configuration of compound **212** at C-2′, the two alkaloids and their congener, psammocindole C (**214**), were synthesized. In addition, enantiomers of psammocindole D (**286**), and four N-lactam analogs, isopsammocindoles A–D (**287** to **290**), were produced. The synthetic scheme was four steps long, and the overall yields were 23%, 21%, and 21% for compounds **212**, **213**, and **214**, respectively (Figure 17). As a result, the R-configuration of compound **212** at C-2′ was assigned [61].

(R)-2-Methylbutan-1-amine was prepared in an 88% enantiomeric excess using a five-step synthetic scheme, while the other amines were obtained commercially. The amines (**270** to **273**) were reacted with ethyl bromoacetate, affording compounds **274** to **277**. Subsequently, further condensation with propionyl chloride produced compounds **278** to **281**, which were used to prepare the *N*-alkyl-α,β-unsaturated γ-lactams (**282** to **285**) by an intramolecular Claisen condensation. Lastly, condensation between the lactams (at C-3) and the indole’s nitrogen afforded compounds **212** to **214** and **286** to **290**, with approximately 30% yields for all analogs [61].

**Figure 18:**
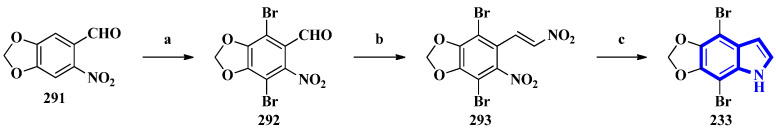
The total synthesis of amakusamine **233**. (a) NBS, conc. H_2_SO_4_, 63%; (b) (1) Al_2_O_3_, CH_3_NO_2_ and (2) Ac_2_O; (c) Iron in AcOH, (last two steps) 89%.

**Figure 19:**
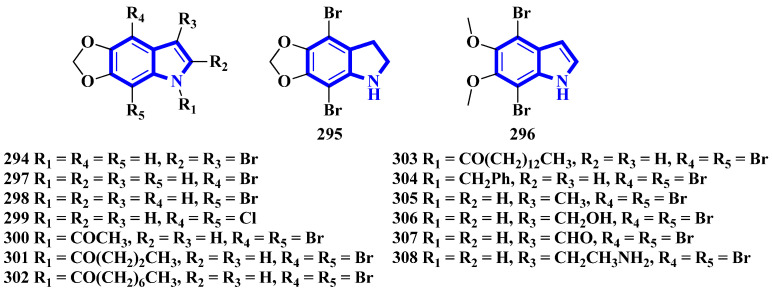
Amakusamine derivatives synthesized **294–308**.

To confirm the structure of amakusamine (**233**), it was synthesized by the same research group that first isolated it. Upon initial failure of a direct debromination of 5,6-methylenedioxyindole, they used an alternative reaction scheme containing three steps and obtained an overall yield of 56% (Figure 18). The synthetic method also enabled the synthesis of 15 synthetic derivatives of compound **233** (**294** to **308**) for an SAR analysis (Figure 19) [66]. The synthesis involved the reaction of 6-nitropiperonal (**291**) with NBS in concentrated H_2_SO_4_, producing **292**. A Henry reaction (using Al_2_O_3_ as a base and nitromethane) followed by dehydration using acetic anhydride transformed **292** into 293. However, since the latter was crystalline and hard to dissolve in many solvents, its crude mixture was reduced directly by an excess of iron in acetic acid, affording compound **233** [66].

**Figure 20:**
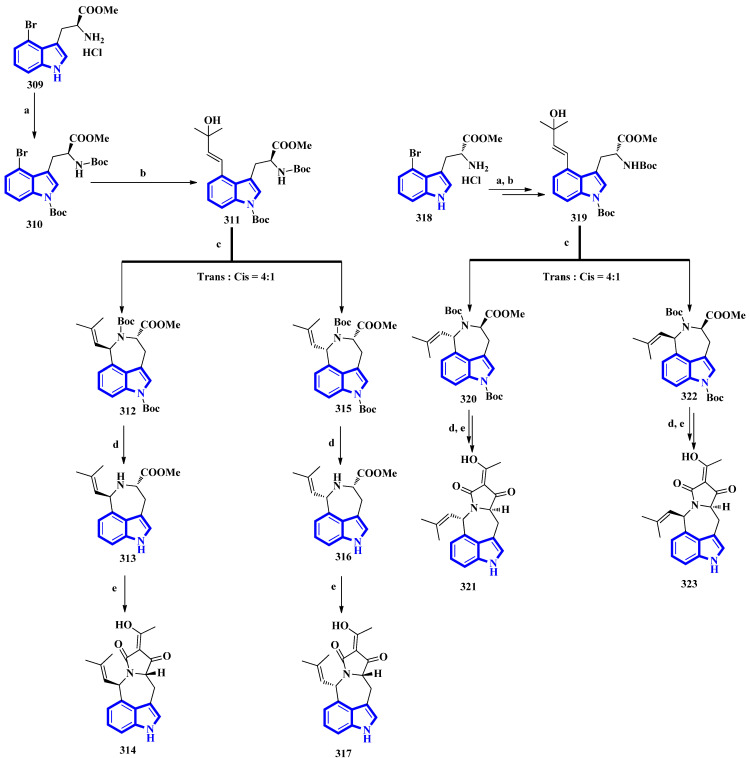
The total synthesis of griseofamine B (**314**) and its isomers **317**, **321**, and **323**. (a) Boc_2_O, Et_3_N, DMAP, DCM, reflux, 2 h, 90%; (b) 2-methyl-3-buten-2-ol, PdCl_2_(PPh_3_)_4_, Ag_2_CO_3_, Et_3_N, 1,4-dioxane, 100 °C, 6 h; 67% (c) PdCl_2_(CH_3_CN)_2_, CH_3_CN, reflux, 2 h; E:Z = 56–58%:15–16% (d) TMSOTf, 2,6-lutidine, DCM, rt, overnight, 87–88%; (e) diketene, Et_3_N, DCM, rt, overnight, 62–63%.

The first total synthesis of griseofamine B (**314**) and its three stereoisomers, 16-epi-griseofamine B, ent-griseofamine B, and 11-epi-griseofamine B (**317**, **321**, and **323**), was carried out in 2022. Compound **314** is an indole-tetramic acid alkaloid isolated from the *Penicillium griseofulvum* fungus in 2018 [86]. In the total synthesis, 4-bromo tryptophan methyl ester hydrochloride was used as the starting reagent, with its l-enantiomer being used to synthesize compounds **314** and **317**. d-Enantiomer 4-bromo tryptophan methyl ester hydrochloride was used to synthesize compounds **321** and **323**. All four compounds were obtained in five steps with yields of 18%, 5%, 19%, and 5%, respectively (Figure 20) [87]. Next, compound **309** was reacted with Boc_2_O, Et_3_N, and DMAP under reflux conditions in DCM to afford **310** in 90% yield. A Heck–Mizoroki reaction of **310** with 2-methyl-3-buten-2-ol in the presence of PdCl_2_(PPh_3_)_4_, Ag_2_CO_3_, and Et_3_N in 1,4-dioxane produced compound **311** in 67% yield. Afterward, refluxing **311** with PdCl_2_(CH_3_CN)_2_ in CH_3_CN generated a pair of diastereomers **312** and **315**. TMSOTf and 2,6-lutidine in DCM were used to remove the Boc groups and gave compounds **313** and **316**. A tandem acylation/Lacey–Dieckmann cyclization of **313** and **316** with diketene was performed to afford **314** and **317**, respectively. Additionally, using the same synthetic scheme with compound **318** as the starting reagent afforded compounds **321** and **323** [87].

### 3.2. Synthesis of Indole Derivatives

**Figure 21:**
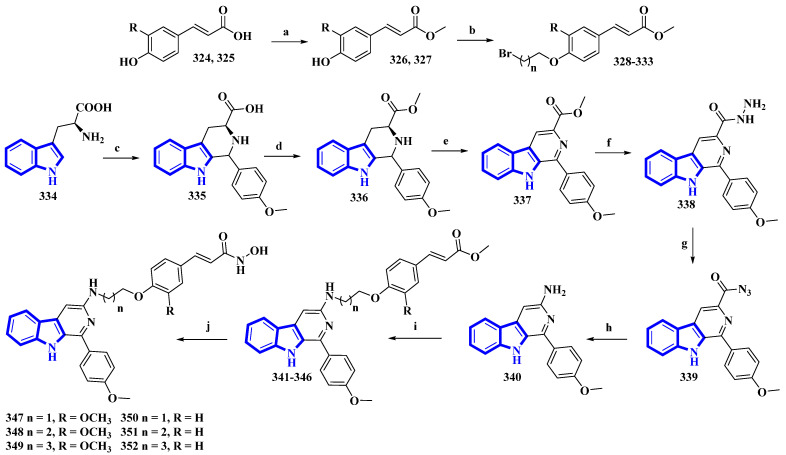
Synthesis of hydroxamic derivatives **347–352**. (a) SOCl_2_, CH_3_OH, 0 °C, 1 h, and then 65 °C, 4 h; (b) ω-Dibromoalkane, K_2_CO_3_, CH_3_CN, reflux, 4 h, 66–75%; (c) 4-methoxybenzaldehyde, AcOH, reflux, 4 h; (d) Thionyl chloride, CH_3_OH, 0 °C, 1 h, and then 65 °C, 6 h; (e) KMnO_4_, DMF, rt, 5 h; (f) Hydrazine monohydrate, CH_3_OH, 50 °C, 6 h; (g) NaNO_2_, HCl; (h) AcOH, H_2_O, 50 °C, 6 h; (i) **328–333**, K_2_CO_3_, CH_3_CN, reflux, 12 h; (j) NH_2_OK, CH_3_OH, rt, 8–12 h, 46–56%.

As β-carboline derivatives generally display only moderate cytotoxicity, new synthetic derivatives were designed to contain a hydroxycinnamic acid moiety inferring HDAC-inhibitory properties that provide synergy and improve their antiproliferative effects [88]. These derivatives (**347** to **352**) differed only in the number of carbons connecting the β-carboline with the hydroxycinnamic acid motif and in the substituents on the phenyl ring in the hydroxycinnamic acid moiety (Figure 21).

(E)-Ferulic acid (**324**) and *p*-coumaric acid (**325**) were esterified using SOCl_2_ in CH_3_OH, yielding compounds **326** and **327**. These compounds were then treated with ω-dibromoalkanes (1,2-dibromoethane, 1,2-dibromopropane, and 1,2-dibromobutane) in the presence of K_2_CO_3_ to produce compounds **328** to **333**. Using a Pictet–Spengler reaction, l-tryptophan (**334**) was transformed into **335** with 4-methoxybenzaldehyde. Compound **335** was then esterified using SOCl_2_ in CH_3_OH, giving compound **336**, which was oxidized using KMnO_4_ in DMF, affording compound **337**. It was then reacted with hydrazine monohydrate to give compound **338** and was converted into **339** using NaNO_2_. Next, compound **339** underwent a Curtis rearrangement to produce compound **340**. Lastly, compounds **328** to **333** were reacted with **340**, yielding residues **341** to **346**, which were then treated with NH_2_OK to produce derivatives **347** to **352**.

**Figure 22:**
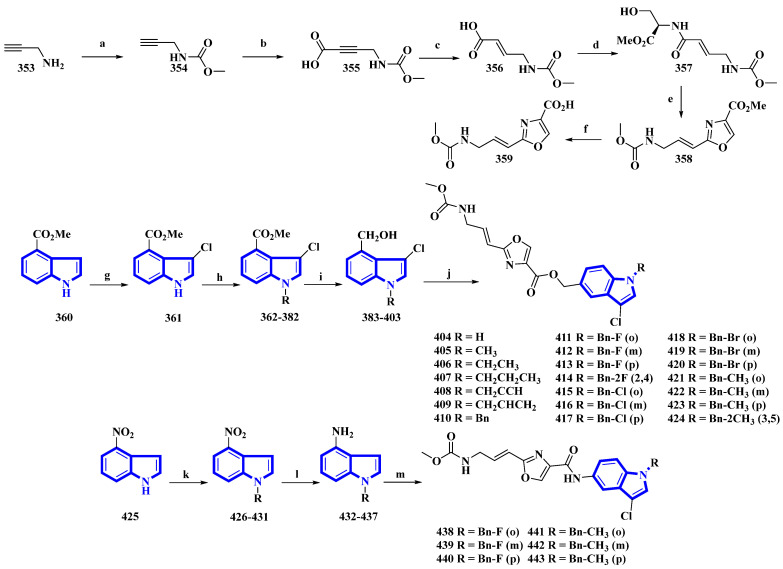
Synthesis of neopeltolide derivatives **404** to **424** and **438** to **443**. (a) NaHCO_3_, 1,4-dioxane, rt, 95%; (b) *n*-BuLi, CO_2_, THF, −78 °C, 84%; (c) H_2_, Lindlar’s catalyst, EtOAc, 91%; (d) l-Serine methyl ester hydrochloride, i-BuOCOCl, *N*-CH_3_-morpholine, THF, 75%; (e) (1) DAST, DCM, −78 °C and (2) BrCCl3, DBU, −20 °C, 62%; (f) LiOH, THF/H_2_O, 88%; (g) NCS, HCl, THF, 82%; (h) Substituted benzyl chlorides, NaH, DMF, rt, quantitative; (i) DIBAL-H, THF, −78 °C, 57–75%; (j) **359**, EDCI, HOBt, Et_3_N, DMF, 43–71%; (k) substituted benzyl bromides, NaH, DMF, rt, quantitative; (l) Fe, NH4Cl, H_2_O, EtOH, reflux, 53–67%; (m) **359**, EDCI, HOBt, DMF, 49–70%.

Neopeltolide, a marine natural product, was isolated from a sponge in the neopeltidae family. It is a highly potent antitumor agent (IC_50_ < 1 nm) and strongly inhibits cytochrome bc1, an essential component of the mitochondrial respiratory chain [89]. Compounds (**404** to **424** and **438** to **443**) were synthesized, replacing its macrolactone ring with an indole having potential as a fungicide. The derivatives were synthesized in two series. The first series (**404** to **424**) contained an ester linkage between the oxazole and indole heterocycles (Figure 22). The second series (**313** to **318**), guided through the bioactivities of the compounds in the first series (Table 2) and the docking studies of leads, replaced the linkage with an amide group and contained only fluorine or methoxy substituents (Figure 22) [90]. The reaction of prop-2-yn-1-amine (**353**) with NaHCO_3_ in 1,4-dioxane affords **354**. Carboxylation of **354** using *n*-butyllithium in THF and a CO_2_ atmosphere produced **355** that was reduced to **356** using Lindlar’s catalyst. Compound **356** reacted with l-serine methyl ester hydrochloride, *N*-methylmorpholine, and isobutyl chloroformate to produce **357**. Compound **357** was reacted with DAST, DBU, and BrCCl_3_ at a low temperature to yield **358**, and LiOH hydrolyzed it to give **359**. Separately, methyl indole-4-carboxylate (**360**) was treated with *N*-chlorosuccinimide in acidic conditions, producing **361**. It was then reacted with substituted benzyl chlorides and NaH in THF, yielding **362** to **382**, which were reduced by DIBAL-H, giving **383** to **403**. Lastly, **359** was reacted with each of the 21 intermediates to give derivatives **404** to **424**. The second derivatives were synthesized by reacting 4-nitroindole (**425**) with substituted benzyl bromides and NaH in anhydrous DMF, yielding **426** to **431**. They were reduced using iron and NH_4_Cl in EtOH and water, affording **432** to **437**. Another time, **359** reacted with each of the six derivatives, producing derivatives of **438** to **443** [90].

**Figure 23:**
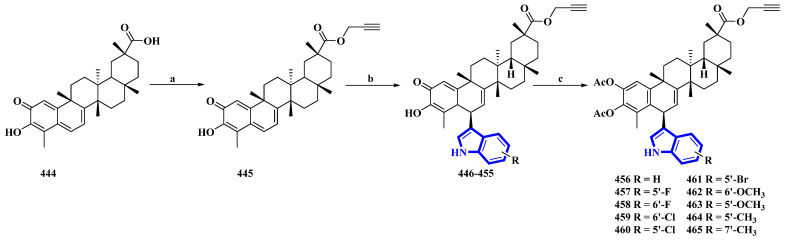
Synthesis of celastrol derivatives **456** to **465**. (a) NaHCO_3_, propargyl bromide, DMF, rt, 78%; (b) substituted indoles, FeCl_3_.6H_2_O, DCM; (c) Ac_2_O, DMAP, DCM, 37–54%.

Celastrol (**444**) is a friedelane-type triterpenoid isolated from *Tripterygium wilfordii* and possesses immunosuppressive properties [91]. However, it has toxic properties. Therefore, its synthetic derivatives were prepared to obtain lower cytotoxicity. Ten celastrol derivatives (**456** to **465**) with indole substituents were synthesized that differed only in the substituents attached to the indole group (Figure 23) [92]. In the synthesis, compound **444** was converted into **445** via a nucleophilic reaction using propargyl bromide and NaHCO_3_ in DMF at room temperature. Compound **445** was transformed using a Friedel–Crafts reaction using substituted indoles and FeCl_3_·6H_2_O in DCM, giving compounds **446** to **455**. These compounds were acetylated using DMAP and Ac_2_O immediately without purification, yielding derivatives **456** to **465**.

**Figure 24:**
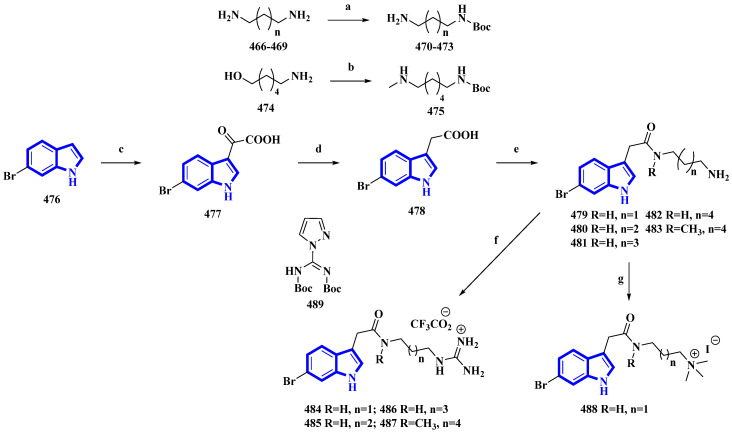
Synthesis of phidianidine A derivatives **479** to **488**. (a) Boc_2_O, rt, overnight, 93–95%; (b) (1) Boc_2_O, Et_3_N, rt, 2 h, (2) MsCl, Et_3_N, 0 °C, 5 h and (3) CH_3_NH_2_, 60 °C, 1 h, 58%; (c) oxalyl chloride, 30 min, 77%; (d) (1) Hydrazine, 80 °C, MW, 15 min and (2) NaOCH_3_, 80 °C, MW, 15 min, 80%; (e) (1) **470–473**, **475**, DIPEA, HATU, 1.5 h and (2) TFA, DCM, 4 h, 8–73%; (f) (1) **479–481**, **483**, **489**, DIPEA, 3 h and (2) TFA, DCM, 2 h, 24–83%; (g) 479, CH_2_O, NaBH_3_CN, AcOH, 21 h and (2) CH_3_I, 0 °C, 53%.

Phidianidine A is a marine natural product isolated from *Phidiana militaris*, a mollusk. The natural product and its synthetic analogs possess both cytotoxic and immunosuppressive activities. However, the antifouling properties of its derivatives were not previously explored despite its resemblance with other potent antifouling MNPs. Therefore, 10 synthetic derivatives (**479** to **488**) having primary amines, guanidines, and a quaternary ammonium compound were produced (Figure 24) [93]. Initially, the diamines **466** to **469** (propan-1,3-diamine, butan-1,4-diamine, and pentan-1,5-diamine) and 6-aminohexanol (**474**) were protected using Boc_2_O. Then, compound **474** was converted into compound **475** using methane sulfonyl chloride and methylamine. Separately, 6-bromoindole (**476**) was converted into derivative **477** using oxalyl chloride. It was then reduced to compound **478** using hydrazine and sodium methoxide. The derivative **478** was then reacted with the diamines **470** to **473** and **475** and deprotected using TFA, affording the primary amines **479** to **483**. Additionally, compounds **479** to **481** and **483** were reacted with DIPEA and **489** to yield guanidine derivatives **484** to **487**. Furthermore, compound **479** was reacted with NaBH_3_CN in formaldehyde and acetic acid, producing quaternary ammonium derivative **488** [93].

### 3.3. Semi-Synthesis of Indole Alkaloids

**Figure 25:**
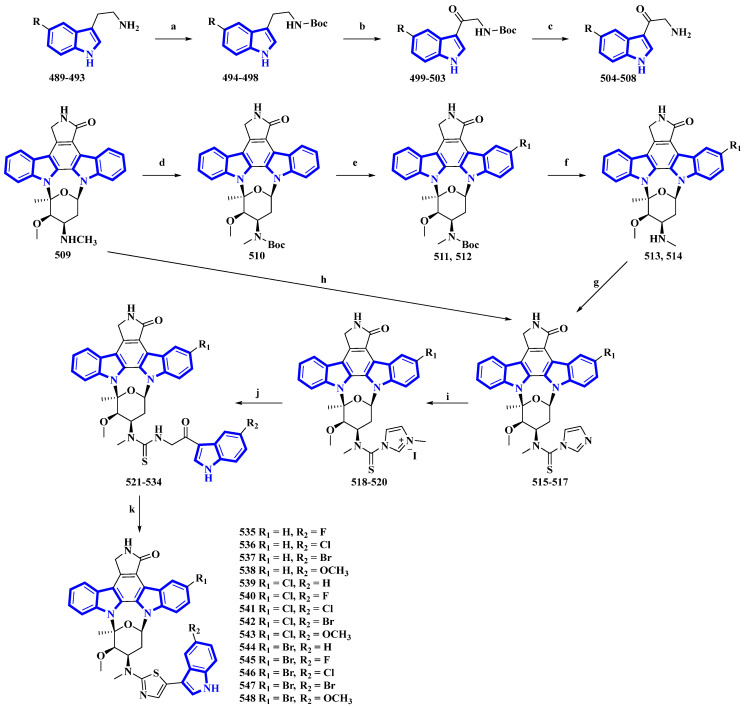
Synthesis of fradcarbazole A derivatives **535** to **548**. (a) Boc_2_O, Et_3_N, THF, 10 °C, 83–99%; (b) DDQ, THF/H_2_O 0 °C, 58–81%; (c) TFA, 10 °C, 72–94%; (d) Boc_2_O, Et_3_N, THF, 0 °C, 76%; (e) NBS, CH_3_OH, DCM, 0 °C, 94%, or NCS, CH_3_OH, DCM, rt, 48%; (f) TFA, DCM, 0 °C, 50–93%; (g) TCDI, Et_3_N, DCM, rt, 71–88%; (h) TCDI, Et_3_N, DCM, rt, 82%; (i) CH_3_I, CH_3_CN, rt, 68–74%; (j) **504–508**, Et_3_N, DMF, rt, 38–56%; (k) (CF_3_CO)_2_O, DCM, EtOH, 0 °C, 47–81%.

**Figure 26:**
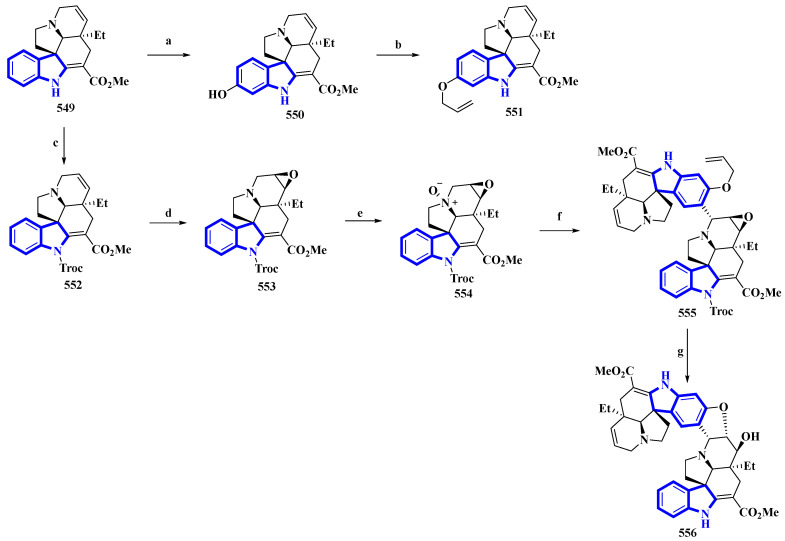
The semi-synthesis of (−)-melodinine K **556**. (a) T16H yeast, 64%; (b) Allyl bromide, K_2_CO_3_, DMF, 68%; (c) NaH, TrocCl, THF/DMF 0 °C, 92%; (d) TFA then *m*-CPBA, DCM, −10 °C then rt, 38%; (e) *m*-CPBA, DCM, 0 °C, 36%; (f) (1) TFAA, DCM, 0 °C to rt and (2) **551**, DCM, 78%; (g) (1) Pd(PPh_3_)_4_, pyrrolidine, DCM, rt and (2) Zn, KH_2_PO_4_, THF, 60 °C, 40%.

Fradcarbazole A, a novel staurosporine-type indole alkaloid containing a thiazole group, was isolated from a mutant strain of *Streptomyces fradiae* and semi-synthesized from staurosporine by the same group [94,95]. To enhance its efficacy as an antitumor agent, 14 derivatives (**535** to **548**) of fradcarbazole A were synthesized by variation only in the substituents on two indole units (Figure 25) [96]. In the synthesis, 5-fluoro/chloro/bromo/methoxy-tryptamines (**489** to **493**) were protected using Boc_2_O to give derivatives **494** to **498** and oxidized using DDQ, producing compounds **499** to **503**. TFA-mediated Boc deprotection resulted in molecules **504** to **508**. Staurosporine (**509**) was protected using Boc_2_O, giving **510**, which was halogenated using NCS and NBS (compounds **511** and **512**), and deprotected by TFA (compounds **513** and **514**). These derivatives and compound **509** were converted to compounds **515** to **517** using TCDI. These compounds were alkylated by CH_3_I in CH_3_CN, affording compounds **518** to **520**. They were then converted into compounds **521** to **534** via a nucleophilic reaction with **504** to **508**. Lastly, intramolecular cyclization reactions of compounds **521** to **534** resulted in **535** to **548** [96].

(−)-Melodinine K (**556**), a complex bisindole alkaloid, was semi-synthesized, using (−)-tabersonine as the starting reagent. The aspidosperma–aspidosperma-type alkaloid was isolated from the *Melodinus tenuicaudatus* plant in 2010. Its cytotoxicity was tested by the same group and was reported to be more potent than cisplatin and vinorelbine in four of the five cancer cell lines [97]. The synthetic route adopted had six steps in its most extended linear sequence (eight steps overall) and afforded compound **556** with a 4% yield (Figure 26) [98]. While planning its synthesis, **556** was divided into two fragments, both of which could be synthesized from (−)-tabersonine (**549**). The total synthesis of **549** was accomplished by the same group in 2013 [99]. However, this precursor was mainly isolated from the seeds of *Voacanga africana* (a small tree). It was bio-transformed into compound **550** using T16H yeast and allylated by allyl bromide and K_2_CO_3_ in DMF, resulting in **551**, the northern fragment of compound **556**. Separately, compound **549** was protected using TrocCl, affording **552**, which was further converted to **553** by TFA and *m*-CPBA. Compound **553** was reacted with *m*-CPBA in DCM, producing its *N*-oxide analog **554**. Compound **554** was treated with TFAA in DCM and coupled with **551** in a Polonovski–Potier reaction, which resulted in **555**. Compound **555** was deprotected by treating with Pd(PPh_3_)_4_, affording **556** [98].

**Figure 27:**
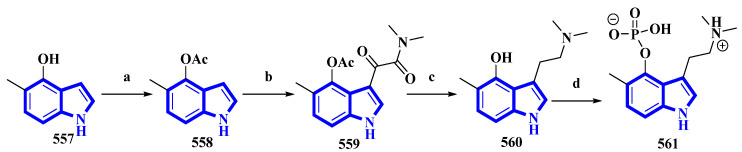
The semi-synthesis of 5-methylpsilocybin 561. (a) Ac_2_O, NaHCO_3_, toluene, 97%; (b) (1) oxalyl chloride and (2) (CH_3_)_2_NH, THF, 77%; (c) LiAlH_4_, THF, 84%; (d) PsiK, ATP, MgCl_2_, H_2_O, 16 h, 44%.

5-Methylpsilocybin (**561**), a novel analog of the psychedelic psilocybin, was produced by enzymatically phosphorylating its synthetic precursor, 5-methylpsilocin (**560**). The 4-hydroxytryptamine kinase (PsiK) enzyme was purified from the *Psilocybe cubensis* fungus. On the basis of the amount of **561** isolated, the overall yield was 28% (Figure 27) [100]. 5-Methyl-1*H*-indol-4-ol (**557**) was acetylated with acetic anhydride and NaHCO_3_ in toluene into compound **558**. It was then treated with oxalyl chloride, followed by dimethylamine in THF, to result in **559**, which, on reduction by LiAlH_4_, resulted in **560**. Compound **560** was incubated with PsiK and ATP for 16 h; the analysis of the mixture by HPLC indicated that 90% of it was successfully converted into 5-methylpsilocybin (**561**) with an overall isolated yield of only 40% [100].

**Table 2:** Bioactivities of all novel indole alkaloids discussed in the article. (a) Tested as a mixture; (b) KB/VJ300 cells grown with vincristine (0.1 μM), which did not affect their growth; (c) methicillin-resistant strain; (d) IC_90_; (e) IC_40_.

Compound	Cytotoxicity (GI_50_) µM [Cell Line]	Ref.
**1**	38 [Huh7], 32 [LN229], 19 [HCT116], 16 [MGC803], 16 [A549], 21 [MDA231]	[20]
**15**	1.1 [KB/S], 4.1 [KB/VJ300 ^b^], 4.7 [PC-3], 4.8 [MCF7], 3.6 [MDA-MB-231], 4.3 [HCT 116], 2.2 [HT-29h]	[22]
**16**	0.1 [KB/S], 1.3 [KB/VJ300 ^b^], 7.9 [PC-3], 9.6 LNCaP, 0.6 [MCF7], 1.4 [MDA-MB-231], 1.1 [HCT 116], 0.3 [HT-29h]	[22]
**17**	0.1 [KB/S], 8.6 [KB/VJ300], 1.5 [KB/VJ300 ^b^], 0.7 [PC-3], 0.3 [MCF7], 0.4 [MDA-MB-231], 0.2 [HCT 116], 0.2 [HT-29h]	[22]
**18**	0.2 [KB/S], 2.6 [LNCaP], 3.5 [MCF7], 5.4 [MDA-MB-231], 3.1 [HCT 116], 2.3 [HT-29h]	[22]
**24**	3.6 [L5178Y], 8.7 [A2780b], 40 [J82], 29 [HEK-293]	[24]
**25**	5.3 [L5178Y], 12 [A2780b], 42 [J82], 22 [HEK-293]	[24]
**26**	5.3 [L5178Y], 28 [HEK-293]	[24]
**27**	12 [A2780b], 55 [J82]	[24]
**28**	32 [A2780b], 100 [J82]	[24]
**29**	8.1 [L5178Y], 7.8 [A2780b], 32 [J82], 37 [HEK-293]	[24]
**37**	6.2 [HT-29]	[27]
**51–54** ^a^	4.1 × 10^−4^ [U87], 7.5 × 10^−4^ [SKOV3], 5.4 [MDA-MB-231 and HCT116]	[30]
**55**	7.3 [HeLa]	[31]
**56**	6.4 [HeLa]	[31]
**71**	0.6 [KB/S], 6.7 [KB/VJ300], 0.2 [KB/VJ300 ^b^], 8.2 [PC-3], 6.2 [LNCaP], 4.5 [MCF7], 4.5 [MDA-MB-231], 0.3 [HT-29], 7 [A549], 7.9 [MRC-5]	[35]
**72**	2.8 [KB/S], 5.9 [KB/VJ300], 6.1 [PC-3], 2 [MCF7], 4.8 [MDA-MB-231], 0.07 [HT-29], 4.2 [HCT 116], 9 [A549], 9.7 [CCD-18Co]	[35]
**73**	1.1 [KB/S], 3.9 [KB/VJ300B], 3.7 [LNCaP], 5.3 [MCF7], 4.8 [MDA-MB-231], 0.07 [HT-29]	[35]
**74**	2.4 [KB/S], 2.9 [KB/VJ300], 3.1 [PC-3], 1.3 [MCF7], 1.4 [MDA-MB-231], 0.03 [HT-29], 2.9 [HCT 116], 6.8 [A549], 4.3 [CCD-18Co]	[35]
**75**	2.5 [KB/S], 5.5 [KB/VJ300], 8.8 [KB/VJ300 ^b^], 7.7 [MCF7], 4.1 [MDA-MB-231], 0.03 [HT-29], 1.5 [HCT 116], 3.9 [A549], 4.1 [CCD-18Co]	[35]
**76**	1.7 [KB/S], 3.6 [KB/VJ300], 4.2 [KB/VJ300 ^b^], 8.5 [MCF7], 0.02 [HT-29], 2.1 [HCT 116], 3 [A549], 5.1 [CCD-18Co]	[35]
**117**	81 [HeLa]	[43]
**118**	42 [HeLa]	[43]
**119**	84 [Huh7], 92 [HeLa]	[43]
**123**	2.45 [A549], 0.96 [Huh7], 0.93 [HeLa]	[43]
**145**	7.5 [SW480]	[48]
**157**	18.7 [HepG2], 28.7 [A-549]	[50]
**160**	17 [U251], 4.5 [U87MG]	[51]
**161**	36 [U251], 43 [U87MG]	[51]
**162**	12 [U251], 2.3 [U87MG]	[51]
**164**	27 [U251], 8.9 [U87MG]	[51]
**166**	36 [U251], 29 [U87MG]	[51]
**167**	19 [U251], 17 [U87MG]	[51]
**168**	12 [U251], 7.4 [U87MG]	[51]
**170**	13 [U251], 11 [U87MG]	[51]
**172**	15 [U251], 19 [U87MG]	[51]
**187**	6.8 [SK-MEL-28], 9.8 [SW480], 6.3 [HepG2], 9.8 [T47D]	[55]
**191**	8.3 [SK-MEL-28], 7.4 [HepG2]	[55]
**192**	6.7 [SK-MEL-28], 7.8 [SW480], 2.5 [HepG2], 8.7 [T47D]	[55]
**193**	9.5 [T47D]	[55]
**211**	5 [KB/S], 7.6 [KB/VJ300], 6.1 [PC-3], 5.9 [MDA-MB-231], 0.2 [HT-29], 4.7 [HCT 116], 2.1 [A549]	[60]
**217**	44.0 [A549], 40.4 [HT-29], 37.3 [HepG2]	[63]
**218**	8.2 [A549], 4.7 [A427], 8.0 [HCT116], 6.7 [HT-29], 6.2 MCF-7, 7.1 [HeLa], 9.1 [HepG2], 44.1 [LO2]	[63]
**219**	13.8 [A427], 38.5 [HCT116], 20.3 [HeLa]	[63]
**220**	18.9 [HeLa], 46.3 [LO2]	[63]
**221**	7.9 [A549], 6.6 [A427], 10.6 [HCT116], 8.2 [HT-29], 7.2 MCF-7, 8.4 [HeLa], 9.6 [HepG2], 35.5 [LO2]	[63]
**222**	9.5 [A549], 4.8 [A427], 12.2 [HCT116], 8.9 [HT-29], 6.5 MCF-7, 8.6 [HeLa], 9.1 [HepG2], 36.3 [LO2]	[63]
**223**	6.9 [A549], 7.5 [A427], 11.2 [HCT116], 8.8 [HT-29], 6.8 MCF-7, 10.1 [HeLa], 10.7 [HepG2], 41.8 [LO2]	[63]
**224**	21.5 [A549], 26.1 [HCT116], 41.5 [HeLa]	[63]
**225**	23.3 [A549], 30.9 [HCT116], 43.8 [HT-29], 44.4 [HepG2]	[63]
**226**	15.9 [A549], 21.2 [HCT116]	[63]
**227**	40.4 [A549], 45.3 [HCT116]	[63]
**239**	1.7 [HeLa], 1.6 [A549], 1.8 [HepG2], 1.5 [SMMC7721]	[70]
**242**	7.9 [HeLa], 7.8 [A549], 8.1 [HepG2], 6.7 [SMMC7721]	[70]
**347**	4 [SMMC-7721], 4.8 [HepG2], 1.8 [Bel7402], 2.8 [Huh7]	[88]
**348**	2.9 [SMMC-7721], 1.4 [HepG2], 1.0 [Bel7402], 1.1 [Huh7]	[88]
**349**	7.1 [SMMC-7721], 5.3 [HepG2], 4.6 [Bel7402], 6.2 [Huh7]	[88]
**350**	6.3 [SMMC-7721], 5.8 [HepG2], 5.2 [Bel7402]	[88]
**351**	4.1 [SMMC-7721], 3.4 [HepG2], 4.0 [Bel7402]	[88]
**352**	9.5 [HepG2], 8.9 [Bel7402]	[88]
**535**	0.51 [MV4-11]	[96]
**536**	0.42 [MV4-11]	[96]
**537**	0.41 [MV4-11]	[96]
**538**	0.36 [MV4-11]	[96]
**539**	0.56 [MV4-11]	[96]
**540**	0.32 [MV4-11]	[96]
**541**	0.59 [MV4-11]	[96]
**542**	0.43 [MV4-11]	[96]
**543**	0.44 [MV4-11]	[96]
**544**	0.36 [MV4-11]	[96]
**545**	0.96 [MV4-11]	[96]
**546**	0.70 [MV4-11]	[96]
**547**	0.39 [MV4-11]	[96]
**548**	0.51 [MV4-11]	[96]
**Antibacterial (MIC; µg/mL)**		
**1**	25 [*B. cereus*], 12.5 [*S. aureus* ^c^]	[20]
**2**	50 [*S. aureus* ^c^]	[20]
**8**	12.5 [*B. cereus*], 25 [*S. aureus*^c^], 25 [*M. lysodeikticus*], 25 [*B. paratyphosum*], 25 [*B. subtilis*], 25 [*E. aerogenes*], 25 [*S. typhi*], 25 [*P. vulgaris*]	[20]
**32**	4 [*B. subtilis*], 16 [*S. typhimurium*], 8 [*M. luteus*], 8 [*M.phlei*]	[26]
**33**	32 [*B. subtilis*], 32 [*S. typhimurium*], 32 [*M. luteus*], 32 [*M. phlei*]	[26]
**40**	8 [*X. o.* pv. *oryzae*], 32 [*R. solanacearum*], 32 [*X. o.* pv. *oryzicola*], 128 [*P. s.* pv. *lachrymans*]	[28]
**42**	32 [*X. o.* pv. *oryzae*]	[28]
**44**	32 [*X. o.* pv. *oryzae*], 128 [*R. solanacearum*], 64 [*X. o*. pv. *oryzicola*]	[28]
**45**	64 [*X. o.* pv. *oryzae*]	[28]
**64**	25 [*H. influenzae* ATCC 4]	[33]
**65**	50 [*H. influenzae* ATCC 4]	[33]
**66**	25 [*H. influenzae* ATCC 4]	[33]
**231**	25 [*M. smegmatis*], 25 [*M. abscessus*], 25 [*M. bovis*]	[65]
**Antifungal (MIC; µg/mL)**		
**1**	6.25 [*A. fragariae*], 25 [*C. cassiicola*], 25 [*A. alternata*], 6.25 [*B. cinereal Pers*], 25 [*C. personata*], 6.25 [*V. dahliaekleb*], 25 [*S. sclerotiorum*]	[20]
**2**	25 [*A. fragariae*]	[20]
**3**	25 [*A. fragariae*]	[20]
**4**	50 [*A. fragariae*]	[20]
**5**	6.25 [*A. fragariae*]	[20]
**6**	25 [*A. fragariae*]	[20]
**7**	6.25 [*A. fragariae*]	[20]
**9**	50 [*A. fragariae*]	[20]
**10**	25 [*A. fragariae*]	[20]
**229**	50 [*F. oxysporum*]	[64]
**Antiplasmodial (IC_50_ µM)**		
**63**	6.1 ^d^ [*P. falciparum*]	[32]
**110**	8.7 [*P. falciparum* FcB1]	[42]
**111**	9.5 [*P. falciparum* FcB1]	[42]
**112**	2.6 [*P. falciparum* FcB1]	[42]
**113**	5.2 [*P. falciparum* FcB1]	[42]
**114**	3.0 [*P. falciparum* FcB1]	[42]
**210**	1.05 [*P. falciparum* FcB1]	[59]
**234**	15.1 [*P. falciparum* 3D7]	[67]
**Antiviral (EC_50_ µM)**		
**109**	70 [HSV-2]	[41]
**137**	5.7 [HCV]	[46]
**196**	7.5 [ZIKV]	[56]
**197**	38 [ZIKV]	[56]
**198**	50 [ZIKV]	[56]
**249**	9.4 [H1N1], 6.7 [RSV]	[72]
**250**	3.7 [H1N1], 2.8 [RSV]	[72]
**Antifouling activity (EC_50_ µM)**		
**486**	2.2 [*A. improvisus*]	[93]
**487**	0.7 [*A. improvisus*]	[93]
**Inhibition of protein phosphatases (IC_50_ µM)**		
**67**	14 [PTP1B], 38 [PTPsigma]	[34]
**68**	27 [PTP1B]	[34]
**70**	23 [PTP1B], 35 [TCPTP]	[34]
**Inhibition of DC secretion of IL-12p40 (% inhibition at 10 µg/mL)**		
**177**	38%	[53]
**185**	36%	[53]
**Promotion of DC secretion of IL-10 (% promotion at 10 µg/mL)**		
**176**	19%	[53]
**Promotion of hBM-MSC secretion of adiponectin (EC_50_ µM)**		
**212**	9.86	[61]
**213**	6.20	[61]
**Inhibition of LPS-induced B-cell proliferation (IC_50_ µM)**		
**237**	0.38	[69]
**238**	47.37	[69]
**Analgesic activity**		
**205**	64.7% (1 mg/kg)	[58]
**206**	50% (5 mg/kg)	[58]
**207**	67.6% (0.04 mg/kg), 76.1% (0.2 mg/kg)	[58]
**208**	55% (5 mg/kg)	[58]
**209**	53% (5 mg/kg)	[58]
**5-HT1A receptor agonist (EC_50_ µM)**		
**83**	10	[37]
**84**	2.2	[37]
**86**	0.1	[37]
**87**	54	[37]
**Inhibition of HDACs (EC_50_ nM)**		
**347**	32 [HDAC1], 125 [HDAC3], 17 [HDAC6]	[88]
**348**	27 [HDAC1], 148 [HDAC3], 13 [HDAC6]	[88]
**349**	61 [HDAC1]	[88]
**350**	75 [HDAC1]	[88]
**351**	57 [HDAC1]	[88]
**352**	125 [HDAC1]	[88]
**Inhibition of porcine SCR (EC_50_ µM)**		
**411**	10	[90]
**438**	1.34	[90]
**439**	1.75	[90]
**440**	1.46	[90]
**442**	0.70	[90]
**Inhibition of acetylcholinesterase (IC50 µM)**		
**98**	10.5	[38]
**Inhibition of quorum sensing (MIC µg/well)**		
**104**	32 [*C. violaceum* CV026]	[39]
**105**	32 [*C. violaceum* CV026]	[39]
**Glucose uptake activity in L6 myoblasts at 50 µM (mmol/L)**		
**203**	5	[57]
**204**	5	[57]
**Triglyceride accumulation promotion in 3T3-L1 cells (EC_50_ µM)**		
**126**	1.03	[44]
**Inhibition of MAO-A (IC50 µM)**		
**127**	0.9 [MAO-A]	[45]
**Inhibition of misc. enzymes (inhibition % at 10 µM)**		
**127**	31 [MAO-B], 21 [BChE]	[45]
**Inhibition of mushroom tyrosinase (IC_50_ µM)**		
**141**	33.2	[47]
**Inhibition of autophagic flux (EC_50_ µM)**		
**145**	20.2	[48]
**Vasorelaxant activity (EC_50_ µM)**		
**158**	2.8	[50]
**159**	2.4	[50]
**Potentiation of imipenem activity (4 ug/mL of imipenem; MIC µg/mL)**		
**173**	8–16 [*S. aureus* ^c^]	[52]
**174**	2–4 [*S. aureus* ^c^]	[52]
**Inhibition of RANKL-induced multinuclear osteoclasts (IC_50_ µM)**		
**233**	10.5 [RAW264]	[66]
**294**	50 ^e^ [RAW264]	[66]
**295**	25.6 [RAW264]	[66]
**296**	6.3 [RAW264]	[66]
**297**	16.8 [RAW264]	[66]
**298**	35.4 [RAW264]	[66]
**299**	40.0 [RAW264]	[66]
**300**	11.7 [RAW264]	[66]
**301**	7.9 [RAW264]	[66]
**302**	11.1 [RAW264]	[66]
**305**	8.1 [RAW264]	[66]
**306**	10.0 [RAW264]	[66]
**307**	13.9 [RAW264]	[66]
**308**	5.9 [RAW264]	[66]

## 4. Bioactivities of Novel Indole Alkaloids

Table 2 only includes the bioactivities of novel indole alkaloids that were isolated, synthesized, and tested from 2019–2022 to enable an extensive structure–activity relationship analysis; the activities of previously reported natural products (which were tested alongside the novel indole alkaloids) are also included. These compounds are referred to by their trivial names. Furthermore, since most articles presented a range of biological activities, we only summarize these activities in this section.

The diterpenoids **1–10** only possessed mild bioactivities. The most potent compound, **1,** showed moderate antifungal and antibacterial activities and weak cytotoxicity. Alkaloids **2–7** and **9–10** showed weak antifungal activities, while **8** displayed moderate antibacterial activity. None of the compounds exhibited inhibitory activity against three pathogenic fungi (*F. oxysporum* Schlecht, *A. solani* Sorauer, and *R. solani*) [20]. Compounds **15–18** displayed potent (sub-micromolar) cytotoxicities against many cell lines, especially against KB/S cells [22]. Compounds **19** to **23** were tested for cytotoxicity against L5178Y cells using the MTT assay. However, all of these were inactive. Previously reported okaramines were also tested, with okaramine H, being the most potent, followed by okaramine G, okaramine A, and okaramine C (IC_50_ = 4.0, 12.8, 13.8, and 14.7 μM, respectively). Additionally, okaramine J was inactive. Hydroxylation at C-2 (**19** vs. okaramine C) or cleavage between 1-N and C-8a (**20** vs. okaramine C) caused a complete loss of cytotoxicity. When an α,α–dimethylallyl group was attached to N-8, the cleavage between N-3′ and C-4′ (okaramine G vs. okaramine A) or reduction of Δ1′ (okaramine A vs. okaramine C) resulted in weak suppression of cytotoxicity. However, when an isoprenyl group was attached at C-7, reduction of Δ1′ (3 or okaramine J vs. okaramine H) completely suppressed cytotoxicity [23]. Compounds **24–29** displayed moderate cytotoxicities against L5187Y cells and weak cytotoxicities against others. Other alkaloids, including 7-hydroxy-13-dehydroxypaxilline, pyrapaxilline, shearinine P, 7-methoxyshearinine P, shearinine Q, and paspalicine were also tested (IC_50_ = 6.2, 10.9, 7.6 μM; latter three inactive). Compound 24’s aromatic ring C was the major determinant in producing its cytotoxicity (compound **24** vs. paspalicine). Additionally, the presence of Δ13 increased the activity of paspalinine derivatives (compound **25** vs. paspalicine) but did not affect the activity of paxillin-type alkaloids. Thus, compound **26** received no benefit from such a motif (compound **26** vs. 7-hydroxy-13-dehydroxypaxilline). Furthermore, the presence of Δ6 slightly enhanced the cytotoxicity of janthitremane derivatives (compound **29** vs. pyrapaxilline). The cleavage of the keto-amide ring also decreased activity (shearinine Q vs. shearinine P). Similarly, replacing a proton at C-7 with a CH_3_ group suppressed activity (pyrapaxilline vs. compound **28** and shearinine P vs. 7-methoxyshearinine P) [24].

Compounds **30** to **31** showed no antifungal, antibacterial, and cytotoxicity activities. A congener, baraphenazine E, exhibited some cytotoxicity and antibacterial activity, indicating that its C-11′-amide may be responsible for its moderately potent activities, as the other alkaloids contained a carboxylic acid group instead. All compounds showed no activity or toxicity in an axolotl embryo tail regeneration assay [25]. The chlorinated cyclic hexapeptides, **32–36**, exhibited no cytotoxicity (tested on HeLa cells). However, compounds **32** and **33** displayed moderate and weak antibacterial activities, respectively, as replacing the phenylalanine residue (**32**) with a leucine residue (**33**) could decrease antibacterial activity. Similarly, replacing the isoleucine residue (**32** vs. **33**) with a valine residue (**35** vs. **36**) completely suppressed activity. Compounds **38** and **39** showed no cytotoxicity, while compound **37** demonstrated moderate cytotoxicity on HT-29 cells [26]. Compounds **41** and **43** showed no antibacterial activity, while compounds **40**, **42**, **44**, and **45** displayed moderate to weak inhibitory activity against *Xanthomonas oryzaepv*. Oryzae (rice bacterial leaf blight). Treatment with compound **40** at 100 µg/mL offered 61% protection, while treatment with 200 µg/mL offered 82% protection from the same pathogen. Furthermore, chaetogline A displayed antifungal properties against *S. sclerotiorum* (EC_50_ = 10.3 μg/mL), while **40** to **45** were inactive. The results indicated that the C-19 carboxylic acid in compound **44** is necessary for its antibacterial activity, while the C-19 ester in chaetogline A is essential for its antifungal activity. Similarly, a second N-methylated indole increased antibacterial activity (**41** vs. **42**) [28].

A mixture of compounds **51–54** was evaluated. It was found to be extremely potent and selective inhibitors of the U87 and SKOV3 cell lines with an EC_50_ of 0.41 nM and 0.75 nM, respectively (SI > 7000; cytotoxicity was also tested against MDA-MB-221 and HCT116 cell lines). The mechanism of action is not proven similar to that of plinabulin (a similar diketopiperazine) due to the nonexistence of vascular disruption and tubulin binding studies [30]. Among compounds **55–62**, only **55** and **56** displayed moderate cytotoxicity, while all other tested indole alkaloids were inactive. Comparing compound **60** with asperversiamide H (both inactive) demonstrated that replacing the C-11 proton with a methoxy group did not significantly affect activity [31]. Compound **63** demonstrated potent antiplasmodial activity, inhibiting more than 90% of liver-stage growth of *P. falciparum* at 6.1 μM. It showed similar antimalarial activity to primaquine [32]. Compounds **64–66** exhibited weak antibacterial activity against the ATCC-4 strain of *H. influenzae*, and compounds **64** and **66** showed slightly more activity than compound **65**. The results indicated that replacing the C-19α proton with a hydroxy group did not alter the bioactivities of (3β,6β,23-trihydroxyolean-12-en-28-oic acid vs. 3β,6β,19α,23-tetrahydroxyolean-12-en-28-oic acid; MIC = 18.8 μg/mL for both), suggesting that it is not a determinant for activities. Additionally, naucleidinal most potently inhibited *H. influenzae* strain ATCC-4 (MIC = 3.1 μg/mL) [33].

Compounds **67–70** are weak inhibitors of PTP1B, while compound **67** is also weakly inhibiting PTP sigma and **70** inhibiting TCPTP. Compound **69** presented no PTP inhibition, and all compounds did not inhibit VHR. Similarly, all four alkaloids did not display any cytotoxicities [34]. Compounds **71–76** showed strong cytotoxicities against multiple cell lines, especially HT-29 (76 IC50: 20 nM) and KB/S (71 IC50: 0.6 μM). Alkaloid **77** was not tested [35].

Compounds **80**, **81**, **82**, **85**, and **88** displayed no activities against the 5-HT1A receptor, a therapeutic target in CNS diseases. Of the remaining four compounds, **83** and **87** displayed weak to moderate activity, while compounds **84** and **86** showed potent agonism. The results demonstrate the importance of stereochemistry in biological systems; **84** and **86** contain a C-16S, whereas their diastereomers **85** and **87** were inactive, having a C-16R configuration. Additionally, a vinyl group at C-20 instead of an ethyl group (**86** vs. **84**) increased bioactivity. Furthermore, docking studies showed that **84** interacted with the 5-HT1A receptor primarily through its two hydrogen bonds between the indole-nitrogen (NH-1) and Asp116, Tyr390. Alternatively, the interaction of compound 86 comprised only one hydrogen bond between the indole-nitrogen (NH-1) and Asn386 [37].

Compounds **89–98** were assessed for cytotoxicity and acetylcholinesterase (AChE) inhibition; none of the alkaloids showed cytotoxicity. Compound **98** moderately inhibited AChE, and docking studies were used to investigate its interactions with the enzyme’s active site. The C-22 carbonyl, N-1, N-4, and the hydroxy group at C-5′ and C-6′ formed hydrogen bonds with Tyr124, Tyr341, Arg296, and His287, respectively, indicating that both the carbonyl group at C-22 and the indole ring’s N-1 were important for its activity [38]. Compounds **99–105** were tested for quorum-sensing inhibition, cytotoxicity, and antibacterial activity. The only bioactive alkaloids were **104** and **105**, which displayed only weak quorum-sensing inhibition in *C. violaceum* strain CV026. Despite stereochemistry being essential in determining bioactivities, enantiomers **103** and **104** displayed the same activities. Additionally, scequinadoline G inhibited quorum-sensing most potently (MIC = 16 μg/well) [39].

Compounds **106–108** showed no cytotoxicity and antibacterial activity, possibly due to their self-fluorescence. Slight modifications such as reducing Δ2 (solitumine B vs. solitumine A; both inactive) or replacing the C-19-bonded NH_2_ with an –OH (106 vs. 107) showed no detectable differences, suggesting that they are not significant determinants of bioactivity [40]. Compound **109** displayed weak antiviral activity against the *herpes simplex* virus 2 (HSV-2). At the same time, other amphiepicoccins, such as amphiepicoccin B, C, D, and H, were also tested for their antiviral and antibacterial (against B. subtilis) activities. Amphiepicoccin C and H displayed weak antiviral activities (IC_50_ = 64 and 29 μM), while amphiepicoccin D and H showed moderate to weak antibacterial activities (MIC = 13 and 25 μM). The rest were inactive. The carbonyl at C-5 is a critical determinant of bioactivity if replacing it with a hydroxy group significantly decreased activity (amphiepicoccin C vs. B). Interestingly, reducing the number of sulfur atoms in the C-2′ to C-7′ sulfide bridge from two to one (amphiepicoccin D vs. H) meaningfully increased antiviral activity but moderately decreased antibacterial activity [41].

Compounds **110–114** displayed moderate to strong antiparasitic activities against the *P. falciparum* strain FcB1. No cytotoxicity was detected against MRC-5 cells. The results showed that the reduction of Δ19′ slightly boosted activity (**111** vs. **110**), but replacing C-22 with a proton significantly suppressed bioactivity (**112** vs. **113**) [42]. Compounds **115–123** were evaluated for cytotoxicity, but only **123** showed potent activity, while **117–119** showed only weak cytotoxicity. Considering compound **123** had a sulfide bridge, compounds **118** and **119** contained an SCH_3_-group at C-6, and compounds **120** to **122** were sulfur-free analogs, especially the sulfide bridge greatly influenced the activity of diketopiperazine [43].

Compounds **124–126** were tested for their ability to promote triglyceride accumulation in 3T3-L1 cells. While compounds **124** and **125** showed no significant activity, compound **126** was a moderate promoter. Additionally, no compound exhibited toxicity against 3T3-L1 cells at 50 µM. Analyzing the structure–activity relationships yielded two important moieties responsible for the bioactivities of compounds: the pyrazino [2,1-b]quinazoline-3,6-dione core and the unsaturated isopropyl group at C-3. Both these motifs are present in scequinadoline D, scequinadoline E (most potent among the compounds tested; EC_50_ = 0.27 and 0.36 µM), while compound **126** only contains the tricyclic core. Suppose the core was replaced with another tricyclic system (quinazoline–pyridine–tetrahydrofuran) or a quinazolinone ring suppressed activity (**124**, scedapin C, and scedapin A; all inactive). Replacing the unsaturated isopropyl group with its saturated analog also reduced activity (**125**, fiscalin C, epi-fiscalin C, scequinadoline A, scequinadoline B, and fiscalin B; all inactive). Furthermore, substituents at C-22 also modulated activity, with ethyl and methyl groups resulting in the most potent analogs (scequinadoline D, scequinadoline E) [44].

Among compounds **127–136**, only compounds **127–129** and **132** were tested for their activities based on chemometric results. Among those three, only compound **127** was active, potently inhibiting MAO-A, while weakly inhibiting MAO-B and BChE. Cimitrypazepine and fargesine were also weak to moderate inhibitors of BChE (18% and 41% inhibition at 10 µM) and potent inhibitors of MAO-A (IC_50_ = 1.4 μM for both). Interestingly, replacing the tertiary amine in cimitrypazepine with an *N-*oxide (as in fargesine) significantly increased BChE inhibition while not affecting MAO-A inhibition. Furthermore, replacing the tertiary amine with a quaternary ammonium ion, as in compound **127**, slightly boosted both BChE and MAO-A inhibition. Conversely, the glucosylated alkaloids **129** and **132** were inactive due to the bulkiness of the glucose group, which hampered the interaction of the alkaloids with the enzyme’s active site [45].

Compound **137** displayed no antibacterial or cytotoxic activities but moderately and selectively inhibited the Hepatitis C virus (SI > 35) [46]. Compounds **138–141** were tested for antibacterial, antifungal activities, and *α*-glucosidase inhibition but were inactive. Compound **141** was weak against mushroom tyrosinases inhibitor, and it is the first example of inhibiting tyrosinases by a thiodiketopiperazine. Changing the steric configuration of C-2 from R to S increased bioactivity (**139** vs. **141**). Additionally, the presence of an OCH_3_/SCH_3_ group at C-2′ and an N-4′-CH_3_ group is necessary for the bioactivities of compounds. Furthermore, a second indole group significantly boosted bioactivity (Leptosin D, IC_50_ = 28.4 μM) [47].

Among compounds **142–150**, only **145** showed moderate cytotoxicity against SW480 while being inactive against other cell lines. The cytotoxicity of tabernaricatine C was also tested, and it potently inhibited HL-60 and SW480 cells (EC_50_ = 3.2 and 3.5 μM). Previously, other alkaloids such as taburnaemine D, tabernaemine I, tabercorine B, tabernaelegantine B, conodurine, and tabernaricatine C (among others), along with compounds **142–150**, were tested for their inhibitory activity against autophagic flux. However, only bisindoles were active, with the most potent inhibitor being taburnaemine D (EC_50_ = 12.9 μM). The linkage pattern of the two indole units might be a crucial factor in determining the activities of the compound [48].

Among compounds **155–159**, only compound **157** showed weak cytotoxicity. The vincamine-type alkaloids **158** and **159** demonstrated vasorelaxant activity. Replacement of the C-11-bonded –OH group in compound 158 with a proton (Δ14-vincamine; EC_50_ = 1.9 μM) or a methoxy group (Δ14-vincine; EC_50_ = 2.2 μM) exhibited slightly increased activity. Interestingly, replacing the same group in its diastereomer **159** with a proton decreased activity (16-epi-Δ14-vincamine; EC_50_ = 3.8 μM) while replacing it with a methoxy group increased activity (16-epi-Δ14-vincine; EC_50_ = 1.9μM). Furthermore, oxidizing C-3 greatly improved vasorelaxant activity (tabersonine vs. 3-oxotabersonine; EC_50_ >10 and 0.8 μM) [50].

Compounds **160–172** displayed weak to moderate cytotoxicities when tested against cell lines U251 and U87MG, and none of them exhibited antimicrobial activities. Nonetheless, results indicated that the methyl substituent on N-1 (the nitrogen of the indole ring) was an important determinant of activity as replacing it with a proton drastically lowered activity (**160**, **162**, or **170** vs. **161**, **163**, or **171**, respectively). Similarly, an OH group at C-13 completely suppressed the cytotoxicity (**164** vs. **165**), and an α-OH group at C-16 deactivated **169** compared to **168**. Furthermore, replacing the C-9 carbonyl with an acetyl group (resulting in the oxidation of the C-9-N-2 bond) meaningfully boosted the activity, which is evident by comparing compound **171** with marine carboline C (EC_50_ = 12, 13 μM against U251, U87MG cells) and to a lower extent **170** with **168**. Furthermore, the steric configurations of the substituents also modulated the activity by replacing the β-OH group on C-16 with an α-OH group, causing a noteworthy increase in cytotoxicity (**166** vs. **167**). Interestingly, changing the geometric configuration of Δ15 from *E*- to *Z*-configuration slightly boosted the potency of methylated-indole analogs (**160** vs. **162**). Nonetheless, non-methylated indole analogs (**161** vs. **163**) were inactive [51].

Compounds **173** and **174** are inactive in vitro antibacterial assays. Nevertheless, they enhanced the activity if co-administered with imipenem (imipenem; MIC = 16–32 μg/mL). Considering that krisynomycin (MIC = 0.25–0.5 μg/mL with 4 μg/mL of imipenem) was more chlorinated than **174**, which was more chlorinated than **173**, the extent of chlorination was thought to be a significant cause of the imipenem-potentiating effects of the compound. Furthermore, chlorination in the alkaloid resulted in increased potentiating effects of imipenem at lower doses [52].

Among compounds **175–185**, only **177** and **185** inhibited the DC secretion of IL-12p40, while **176** increased the secretion of DC cytokine IL-10. Compounds **177** and **185** did not affect the viability of DCs, demonstrating that the decrease in secretion was not due to cell damage. The results indicated that the nature of the C-14 substituent did not affect bioactivity noticeably. Replacing a proton with a hydroxy (**180** or **182** vs. **181** or **183**) or methoxy (**182** vs. **184**) did not meaningfully affect activity. Furthermore, pyrrolo [2,3-b]indole (180 to 184) and oxindole (**178** and **179**) alkaloids were found to be inactive. On the other hand, 6-bromoindole alkaloids (**177** and **185**) were active, suggesting that modified indoles decreased the bioactivities of compounds [53]. Compound **186** demonstrated no antiplasmodial activity against the ring stage of the *P. falciparum* strain 3D7 nor the *P. falciparum* strain Dd2. It was non-cytotoxic against HEK293 cells, too [54].

The cytotoxicities of compounds **187–195** against various cell lines were determined. Molecules **187**, **191**, **192**, and **193** exhibited moderate cytotoxicities. The only difference between **190** and **191** was the oxidation of C-18, suggesting that the absence of the carbonyl group significantly increases the compound’s activity. Furthermore, replacing a proton with a hydroxy group at C-2 causes a significantly suppressed activity (**192** vs. **191**). Stereochemistry (**192** vs. **194** and **193** vs. **195**) was also a foremost determinant of the activities of alkaloids; switching C-14′ from an S to R configuration significantly reduced their activities [55].

Compounds **196–200** were not cytotoxic against the A549 cell line. Similarly, only **196**, **197**, and **198** inhibited the Zika virus. Previously reported activities of natural products suggested that the F-ring was a significant factor of antiviral activity; an open F-ring afforded an inactive compound (emindole SB), while a furan F-ring yielded the most potent compound in the series, JBIR-03 (EC_50_ = 4.2 μM). However, compared to JBIR-03, the weaker activities of fusaindoterpene A (EC_50_ = 12 μM) and asporyzin A (EC_50_ = 18 μM) indicated that B- and C-rings also modulate bioactivity. Furthermore, adding an oxygen atom into the eight-membered ring of asporyzin A (as in fusaindoterpene A) slightly increased activity [56].

Compounds **201–204** were tested against the HEI-OC1 cell line to determine their protecting ability. Compound **201** displayed potent protecting effects (optical density = 0.7, compared to the blank control’s 0.5), while the other compounds were inactive. The alkaloids were also tested to determine if they stimulated glucose uptake in cultured rat L6 myoblasts. Only compounds **203** and **204** promoted glucose uptake in the myoblasts. Among the non-novel natural products tested, beauverolide Ka displayed both protective ability (optical density = 0.69) and promotion of glucose uptake (9 mmol/L). The results indicated that a hydroxy substituent on C-13 (para-substituent of the phenyl ring) instead of a proton decreased protective ability significantly (beauverolide Ka vs. **203** and **201** vs. **204**). Interestingly, replacing the indole ring, the C-2 substituents, or the C-13 proton in beauverolide Ka drastically decreased its glucose uptake promotion activity, evident when comparing it to beauveamide E (inactive), **201**, and **203** [57].

The analgesic effects of compounds **205–209** were studied, with compound **207** exhibiting more potent analgesic activities than morphine at 0.04 and 0.2 mg/kg doses. Since **205** was more active than humantenine N_4_-oxide and humantenine, its oxazolidinone ring may enhance bioactivity. Conversely, compound **207** and *N*-desmethoxyhumantenine were more potent than alkaloids with an N-1-OCH_3_ motif (**206**, humantenine N_4_-oxide, 11-hydroxyhumantenine, humantenine, and humantenirine), indicating that the group might cause a decline in activity. Moreover, compound **208** displayed lower activity than epi-koumidine and koumidine, inferring that the N_4_-oxide (not possessed by the latter two) decreased the activity of sarpagine-type alkaloids. Furthermore, compound **206**, 11-hydroxyhumantenine, and humantenirine contained a hydroxy or methoxy substituent at C-11and showed less potent activities, suggesting that these groups decreased the activities of humantenine-type alkaloids [58]. Compound **210** demonstrated no cytotoxicity against the MRC-5 cell line; however, it potently inhibited *P. falciparum* strain FcB1. Comparing its antiplasmodial activity with vobasine (IC_50_ = 22.5 μM) revealed that the C-3 bonded –SH group is necessary for the activity of vobasane-type alkaloids; nevertheless, vobasine contains a carbonyl. Additionally, the discovery of compound **210** is of particular biosynthetic interest, as it was proposed to be a step in forming the ion brunonines A and B [59].

Compound **211** displayed moderate to robust cytotoxicities against various cell lines and selectivity (SI > 5) when tested against HT-29 and CCD-18Co cells. It induced mitochondrial apoptosis, and in vitro tubulin polymerization assays confirmed it as a microtubule-stabilizing agent. It was also predicted to bind at the taxol-binding site in β-tubulin. The docking models showed that compound **211** had several nonpolar interactions and hydrogen bonds with the amino-acid residues in the binding pocket, resulting in its calculated interaction strength being more significant than paclitaxel, which was consistent with the observed microtubule-stabilizing activity. Compound **211** also satisfied the criterion of drug-likeness, with a score of 3.28 (vincristine 4.03, Taxol 0.82), well within the proposed acceptable range of 2–7 [60,101].

The adiponectin secretion-promoting activities of compounds **212–214** and their synthetic isomers, **286–290**, were investigated in hBM-MSCs. However, the EC_50_ of compounds **214** and **286–290** was greater than 10 μM. Compounds **212** and **213** were more potent than bezafibrate and increased lipid accumulation in differentiated adipocytes. The results suggested that they improved insulin sensitivity (like pioglitazone). Comparing the activities of the alkaloids demonstrated the importance of handedness in biological systems; enantiomers of the compound **212** and **286** displayed significantly less potency. A bulky phenyl group at C-2′ may suppress bioactivity by hindering the interactions of compounds with the target’s active sites (**213** vs. **214**) [61].

Docking studies showed the indole acetonitrile in compounds **215** and **216**, and other compounds such as 3-indole acetonitrile and 4-methoxy-3-indole acetonitrile, which strongly bind with the HA and form hydrogen bonds with the THR59F and GLU90D residues. This hydrogen bonding possibly modulated the function of HA and produced the supposedly antiviral activities of compounds. Nevertheless, despite reporting compound **215** as an inhibitor of HA, no quantitative information is available [62].

Compounds **217–227** displayed weak to moderate cytotoxicities against various cell lines, while compound **228** was inactive. Western blot analysis showed that compound **218** induced apoptosis of MCF-7 cells via the Bcl-2/caspase-3/PARP pathway. Results revealed that reducing Δ19 caused a decrease in cytotoxicity (**218** vs. **219** and **223** vs. **220**), which infers that structural rigidity is required to exhibit cytotoxicity. Interestingly, the geometric conformation of Δ19 did not significantly influence activity, as evident when comparing the activities of the Z-isomer (271) and the E-isomer (**272**) [63]. No cytotoxicity or antibacterial activity was detected when compounds **229** and **230** were tested. Compound **229** displayed weak antifungal activity against *F. oxysporum* [64]. Compounds **231** and **232** showed no cytotoxicity, and only compound **231** showed weak antimycobacterial activity. Replacing the C-12 proton in compound **231** with a methoxy group as in **232** completely lost the activity [65].

Compound **233** and its novel synthetic derivatives (**294–308**) were tested for their inhibition of RANKL-induced multinuclear osteoclasts in RAW264 cells. The weaker activity of **295** compared to compound **233** demonstrated the importance of Δ2 for bioactivity. If the methylenedioxy ring was replaced with the two methoxy groups, as in **296,** slightly enhanced activity was observed. The results also indicated that the presence and positions of bromine atoms are a significant determinant of bioactivity as 5,6-methylenedioxyindole. Shifting the position of bromines as in **294** or replacing them with chlorines as in **299** significantly suppressed the activities. While the bromine at C-4 was believed essential for activity, the bromine at C-7 slightly enhanced activity when C-4 was unsubstituted (**298** compared to 5,6-methylenedioxyindole). Moreover, the presence of chlorines slightly improved potency when bromines were absent (**299** versus 5,6-methylenedioxyindole). Similarly, *N*-acyl derivatives were used to study the effects of varying chain lengths on activity. Compounds **300–302** with C2–C8-alkyl chains showed activity, but compounds **303** (C14-alkyl chain) and **304** (benzoyl derivative) showed no activity, demonstrating that long or bulky groups cause a decline in activity. Furthermore, the effects of substituents at C-3 were studied by comparing the activities of compounds **305**, **306**, and **307**, which contained methyl, hydroxymethyl, and formyl substituents, respectively. Although compounds **305** and **306** had similar activities as **233**, compound **307** was less potent. Compound **308**, a tryptamine derivative, was more potent but was found cytotoxic at 25 μM, while none of the other derivatives showed toxicity even at 50 μM [66].

Compound **234** displayed weak antiplasmodial activity against *P. falciparum* strain 3D7 and very weakly inhibited the growth of HEK293 cells (32.5% inhibition at 40 μM) [67].

While compounds **235** and **236** were not tested, other compounds—(1*H*-indol-3-yl)-oxoacetamide, and indole-3-aldehyde—were also produced by *E. coli* harboring the EDB cluster and were examined for their ability to repel *C. elegans*. The two molecules had a chemotaxis index of −0.18 and −0.08 at 0.2 mM, implying moderate repulsion and confirming that *P. fluorescens* NZI7′s EDB cluster enables it to convert external tryptophan into indole-containing molecules that repel their predators, *C. elegans* [68]. Compound **237** (and, to a much lesser extent, **238**) inhibited LPS-induced B-cell proliferation, with the former displaying high selectivity (SI > 548). The results indicated that the C-6 methoxy group of compound **237** is essential for activity. Replacing it with a proton, as in 238, led to an increase in toxicity and a sharp decline in inhibitory activity. Compound **237** was also inactive against the four tyrosine kinases ibrutinib (the positive control) inhibited, suggesting that compound **237** exhibited its activity via a new mechanism. However, neither compound inhibited concanavalin-A-induced T-cell proliferation [69].

Among compounds, **239–244**, **239** and **242** exhibited cytotoxic effects, indicating that the N-OH group was a key determinant of activity (**239** compared to **240**). Similarly, compound **242** had more potent activity than compound **241**, suggesting that the more rigid cyclized form of compound **242** improved the cytotoxicity. Molecule **239** also caused mitochondrial dysfunction in HeLa cells, and compound **239**-induced cell apoptosis was related to generating reactive oxygen species in HeLa cells. Furthermore, compound **239** increased the phosphorylation levels of JNK, ERK, and p38 in HeLa cells, suggesting that compound **239** activated the MAPK signaling pathway, which helps mediate cell proliferation and apoptosis [70,102].

The hepatoprotective activities of compounds **245–248** were assayed in transgenic fluorescent zebrafish with liver injury. Quantitative analyses showed that 20 μM of compounds **245**, **246**, and **248** exhibited moderate hepatoprotective activities equal to those of the positive control (50 μM S-adenosyl-l-methionine). In addition, C-3 substituents were also a significant determinant of bioactivity as replacing the 2-aminoethanal group in **248** with a methyl formate group (as in **247**) or with a 2-aminopropanoic acid group (as in C-mannosyl tryptophan) resulted in inactivity [71].

Although compounds **249** and **250** displayed moderate to strong antiviral activities, they were toxic against MDCK and Hep-2 cells with CC_50_ values ranging from 13 to 31 μM, drastically lowering their therapeutic ratios. Interestingly, the hemiketal form (**250**) of compound **249** displayed more potent antiviral activities while also being more toxic [72].

The antibacterial activities of compounds **314**, **317**, **321**, and **323** were evaluated but were considerably less potent than their congener, griseofamine A’s activity (MIC = 8–16 µg/mL). Since all compounds tested contained the 3-acyltetramic acid moiety, the putative notion that the motif was a major determinant of antibacterial activity was not supported [87]. Compounds **347–352** displayed moderate to strong cytotoxicities against various cell lines. The results indicated that increasing the length from four to five (counting the heteroatoms) of the linker chain increased cytotoxicity (**347** vs. **348** and **350** vs. **351**). However, a further increase (from five to six) suppressed bioactivity (**348** vs. **349** and **351** vs. **352**). Replacing the methoxy group in the trisubstituted phenyl ring with a proton weakened the activities of derivatives (**347**, **348**, and **349** vs. **350**, **351**, and **352**) [88]. Moreover, compound **348** displayed the most robust cytotoxicity in the series while exhibiting minimal toxicity on normal hepatic LO2 cells (showing high selectivity). It also inhibited HDAC1 and HDAC6 while enhancing the acetylation levels of histone H3 and α-tubulin. In addition, it induced more significant cancer cell apoptosis than SAHA by regulating the expression of apoptotic proteins Bax, Bcl-2, and caspase 3. Compound **348** also induced important autophagic flux activity in Bel7402 cells by increasing the expression of Beclin-1 and LC3-II proteins and suppressing LC3-I and p62 production. Furthermore, compound **348** also significantly inhibited PI3K/Akt/mTOR signaling, an essential cell-growth-promoting pathway aberrantly activated in many cancers [88,103].

Compounds **404–424** and **438–443** were evaluated for their inhibitory activity against porcine SCR (mixture of bc1 and complex II). The first series (**404–424**) showed weak activities, except **411,** only inhibiting 10–49% of SCR at 10 µM. The binding of the lead compound **411** of the first series was analyzed with the complex. The carboxyl oxygen in carbamate formed an H-bond with Glu271. However, the indole and phenyl rings were almost perpendicular with Phe274, resulting in weak interactions with the residue. Previous studies by the same group showed that the π–π interactions between neopeltolide and Phe274 were essential for activity [104]. Furthermore, the carboxyl oxygen in the ester did not form an H-bond with His161, also an essential factor for bioactivity [104]. The second series (**438–443**) was synthesized with an amide linker to improve the interactions between the derivatives and key residues. Similarly, it contained only fluorine- and methyl-substituted benzyls as substituents on the indole ring (first series derivatives with such substituents were generally more potent). These modifications considerably increased the potency of the second series derivatives. Again, the binding mode of the most potent derivative **442** was analyzed. It formed π–π interactions with Phe274 and an H-bond with Glu271 and His161. Interestingly, the two positional isomers of compound **442** (which differed in the positions of substituents on the benzyl group), **441** (*o*-methyl), and **443** (*p*-methyl) were considerably less potent, demonstrating that *m*-methyl was the most bioactive configuration. In contrast, for the fluorine substituents, *o*-fluorine was the most bioactive configuration (**438** vs. **439** vs. **440**) [90].

None of the celastrol-indole derivatives (**456–465**) inhibited concanavalin A-induced T-cell proliferation or displayed cytotoxicities. [92] Phidianidine A and its synthetic structure variants (**479–488**) were screened against the settlement and metamorphosis of *Amphibalanus improvisus* cyprids, with compounds **486** and **487** exhibiting the most potent activities. It demonstrated that only the guanidine derivatives showed significant activity, while primary amine (**479–483**) or quaternary ammonium **488** derivatives were relatively inactive. Additionally, increasing chain length increased bioactivity among the guanidine derivatives (**484–487**). Furthermore, since both compounds 486 and 487 were more potent than phidianidine A (EC_50_ = 4.0 µg/mL), the original scaffold’s transformation into a simpler, more bioactive structure that did not contain the constrained 1,2,4-oxadiazole ring was deemed successful [93].

Compounds **535–548** displayed potent cytotoxicity against the MV4-11 cell line. In addition, compound **540** was the most powerful derivative in the series and induced apoptosis of MV4-11 cells. It also arrested cells in the G0/G1 phase and reduced the expression of FLT-3, CDK2, and c-kit. The preliminary structure–activity relationship study yielded interesting results: if the substituent on C-3 was a proton (**535–538**), the most activity-boosting substituent on C-5ʹʹ was a methoxy group (**538**). Additionally, if C-3 was bonded to chlorine (**539–543**), then fluorine on C-5ʹʹ produced **540** (the most potent derivative overall). Lastly, for bromine on C-3 (**544–548**), a proton on C-5ʹʹ afforded the most potent bromine-containing derivative **544** [96]. However, compound **561** was tested for psychedelic-like activity using the mouse head-twitch response assay, which showed that it was more potent than dimethyltryptamine but less potent than psilocybin [100].

## 5. Conclusions

The 354 novel indole alkaloids discussed—consisting of 250 isolated and 87 synthesized—represent only a tiny fraction of the known indole-alkaloids, according to both the number and the structural diversity of predicted indole-containing compounds yet to be discovered. This massive gap between known and predicted indole alkaloids and the group’s potent, selective, and variegated bioactivities represents a worthwhile opportunity for chemists, who can leverage modern spectroscopic and biological tools to determine the alkaloids’ structures and mechanisms of action efficiently. This article described recent advances in the field to draw further attention to unique alkaloids that either possess unprecedented motifs or display substantial activities against their targets and to provide a convenient, easily accessible list of information regarding the isolations and bioactivities of alkaloids. Similarly, reappraisals were included to demonstrate the efficacy of modern-day spectroscopic techniques. At the same time, syntheses (both total and semi) were discussed to highlight the second aspect of drug development: optimizing the lead.

## Figures and Tables

**Table 1:** Sources and isolation procedures of novel indole alkaloids (**1–250**).

Compound Serial	Source	Isolation Method	Reference
**1 to 10**	*Tolypocladium*	Extracted with DCM/CH_3_OH (1:1); residue suspended in water and extracted with EtOAc	[20]
**11 to 14**	Didemnidae, Eudistoma, Pseudodistoma	Extracted with CH_3_OH, followed by DCM	[21]
**15 to 18**	*Tabernaemontana corymbosa*	Extracted with EtOH, concentrated, and partitioned with 3% tartaric acid	[22]
**19 to 23**	*Aphanoascus fulvescens*	Extracted with EtOAc	[23]
**24 to 29**	*Penicillium*	Extracted with EtOAc; residue extracted with hexane/CH_3_OH (90%)	[24]
**30 to 31**	*Streptomyces* sp. PU-10A	Extracted with CH_3_OH; mixed with 3% (*w/v*) XAD-16 resin, filtered, washed with water, and extracted with CH_3_OH	[25]
**32 to 36**	*Streptomyces noursei*	Extracted with CH_3_OH; the aq. layer was freeze-dried	[26]
**37 to 39**	*Kopsia arborea*	Extracted with CH_3_OH, concentrated, and partitioned with 3% tartaric acid	[27]
**40 to 45**	*Chaetomium globosum*	Extracted with EtOAc	[28]
**46 to 50**	*Alstonia balansae*	Basified with NH_4_OH (6 M) and extracted with DCM; extracted using HCl (0.1 M); basified with NH_4_OH (pH = 10) and extracted with DCM	[29]
**51 to 54**	Actinomycete AJS-327	Extracted with EtOAc	[30]
**55 to 62**	*Aspergillus versicolor*	Extracted with 95% EtOH; residue suspended in water and extracted with EtOAc (1:1 *v/v*)	[31]
**63**	Inflatella	Extracted with DCM: CH_3_OH (1:1), followed by CH_3_OH: H_2_O (1:1)	[32]
**64 to 66**	*Nauclea latifolia*	Extracted with CH_3_OH; residue suspended in 5% H_2_SO_4_ and extracted with DCM; solution neutralized with NH_3_ and extracted with DCM	[33]
**67 to70**	*Penicillium*	Extracted with EtOAc	[34]
**71 to 77**	*Alstonia penangiana*	Extracted with CH_3_OH, concentrated, and partitioned with 3% tartaric acid	[35]
**78 to 79**	*Uncaria longiflora* var. pteropoda	Extracted with CH_3_OH, followed by trituration using hexane, CHCl_3_, EtOAc, and CH_3_OH; CHCl_3_ extract was acidified (5% HCl) and filtered; the filtrate was basified with 37% NH_4_OH	[36]
**80 to 88**	*Uncaria rhynchophylla*	Extracted with 95% EtOH and 75% EtOH; residue suspended in water and extracted with DCM and *n*-BuOH	[37]
**89 to 98**	*Uncaria rhynchophylla*	Extracted with 95% EtOH, 75% EtOH, and 50% EtOH; residue dissolved in 4% HCl and filtered; filtrate basified with NH_3_. H_2_O to pH 9–10 and partitioned sequentially with CHCl_3_ and EtOAc; remaining alkaline soln. was neutralized with HCl and subjected to microporous resin HP-20	[38]
**99 to 105**	Aspergillus	Extracted with EtOAc	[39]
**106 to 108**	*Penicillium solitum*	Extracted with EtOAc; residue dissolved in H_2_O:CH_3_OH (5:95; *v*/*v*) and partitioned with hexane; aq. fraction was extracted by a 1:1:1 mixture of XAD 2, 4, and 7; water-soluble organic material was desorbed with CH_3_OH and CH_3_OH/acetone (1:1; *v*/*v*)	[40]
**109**	*Epicoccum nigrum*	Supernatant extracted with EtOAc; mycelia were macerated and extracted with acetone	[41]
**110 to 114**	*Picralima nitida*	Extracted with EtOH	[42]
**115 to 123**	*Chaetomium cochliodes*	Extracted with DCM/CH_3_OH (1:1); residue dispersed in water and extracted with DCM and EtOAc	[43]
**124 to 126**	*Scedosporium apiospermum*	Extracted with CH_3_OH	[44]
**127 to 136**	*Psychotria nemorosa*	Extracted with CH_3_OH; suspended in 1 M HCl and partitioned with DCM; aq. phase was basified using NH_4_OH (pH = 9.5) and partitioned with DCM	[45]
**137**	*Penicillium raistrickii*	Extracted with EtOAc	[46]
**138 to 141**	*Phaeosphaeria fuckelii*	Extracted with EtOAc	[47]
**142 to 150**	*Tabernaemontana corymbosa*	Extracted with CH_3_OH; solution acidified with saturated tartaric acid (pH = 2–3) and extracted with EtOAc; aq. phase basified using saturated NaOH and extracted with DCM	[48]
**151 to 154**	*Mitragyna speciosa*, Havil. Rubiaceae	Extracted with CHCl_3_:CH_3_OH (1:1) and 10% KOH by maceration; residue dissolved in 1 M HCl: hexane (1:1); aq. phase basified using conc. NH_4_OH (pH = 9) and extracted with CHCl_3_	[49]
**155 to 159**	*Melodinus hemsleyanus*	Extracted with 95% EtOH; suspended in water and acidified using 5% HCl (pH = 2–3); partitioned with CHCl_3_; aq. phase basified using 10% NH_3_ soln. (pH = 9–10) and extracted with CHCl_3_	[50]
**160 to 172**	*Actinoalloteichus*	Extracted with EtOAc	[51]
**173 to 174**	*Streptomyces canus*	Extracted with acetone	[52]
**175 to 185**	*Flustra foliacea*	Extracted with CH_3_OH, DCM; residue suspended in water and partitioned with EtOAc; organic fraction suspended in 90% CH_3_OH and partitioned with hexane; CH_3_OH fraction suspended in 60% CH_3_OH and partitioned with DCM	[53]
**186**	*Amathia lamourouxi*	Extracted with CH_3_OH	[54]
**187 to 195**	*Tabernaemontana pachysiphon*	Extracted with CH_3_OH, and acidified using saturated tartaric acid (pH = 2–3); extracted with EtOAc; aq. phase basified using saturated NaOH (pH = 9–10) and extracted with DCM	[55]
**196 to 200**	Fusarium	Extracted with EtOAc	[56]
**201 to 204**	Beauveria	Extracted with EtOAc	[57]
**205 to 209**	*Gelsemium elegans*	Extracted with 95% EtOH; suspended in water, acidified using 0.3 M HCl (pH = 3), and partitioned with CHCl_3_; aq. phase basified using NH3 soln. (pH = 10) and extracted with CHCl_3_; aq. extract partitioned with *n*-BuOH	[58]
**210**	Mostueabrunonis	Alkalinized with NH_4_OH (25%) and extracted with EtOAc; extracted with 1% HCl, and basified using NH_4_OH (pH = 10); extracted with EtOAc	[59]
**211**	*Alstonia penangiana*	Extracted with CH_3_OH, concentrated, and partitioned with 3% tartaric acid	[60]
**212 to 214**	*Psammocinia vermis*	Macerated and extracted with CH_3_OH and DCM; partitioned between H_2_O and *n*-BuOH; latter fraction was repartitioned between H_2_O-CH_3_OH (15:85) and hexane	[61]
**215 to 216**	*Isatis indigotica*	Submerged in 2.5% ammonia for 6 h and extracted with 80% and 70% ethanol; concentrated and extracted with ethyl acetate	[62]
**217 to 228**	*Pseudeurotium bakeri*	Extracted with EtOAc	[63]
**229 to 230**	*Clonostachys rosea*	Macerated culture extracted with acetone/DCM/CH_3_OH; residue partitioned with petroleum ether, EtOAc, and *n*-BuOH	[64]
**231 to 232**	*Voacanga africana*	Extracted with CH_3_OH; suspended in CH_3_OH: H_2_O (10%) and basified using NH_3_ (pH = 12); extracted with DCM	[65]
**233**	Psammocinia	Extracted with EtOH and CH_3_OH; residual solution extracted with EtOH; fraction partitioned between hexane and 90% CH_3_OH: H_2_O	[66]
**234**	*Synoicum prunum*	Extracted with CH_3_OH	[67]
**235 to 236**	*Escherichia coli*	Extracted with EtOAc	[68]
**237 to 238**	*Ophiorrhiza japonica*	Extracted with 90% CH_3_OH; residue dissolved in 0.5% aq. HCl and extracted with EtOAc; aq. phase basified using 10% NH_4_OH (pH = 8–9) and extracted with EtOAc	[69]
**239 to 244**	*Aspergillus sclerotiorum*	Extracted with EtOAc; residue dissolved in CH_3_OH: H_2_O (9:1) and partitioned with cyclohexane	[70]
**245 to 248**	*Neopetrosia chaliniformis*	Extracted with CH_3_OH	[71]
**249 to 250**	*Penicillium oxalicum*	Extracted with EtOAc	[72]

## Data Availability

Not applicable.

## Abbreviations

**WHO**	World Health Organization
**LSD**	Lysergic acid diethylamide
**DCs**	Dendritic cells
**HDAC**	Histone deacetylase
**MIAs**	Monoindole alkaloids
**CANPA**	Computer-Assisted Natural Products Anticipation
**ETP**	Epipolythiodioxopiperazine
**HMDA**	Hydroxy-4-methyldecanoic acid
**EDB**	Edible
**LCPA**	Long-chain polyamine
**DFT**	Density-functional theory
**THF**	Tetrahydrofuran
**TBAF**	Tetra-n-butylammonium fluoride
**RANKL**	Receptor activator of nuclear factor kappa-Β ligand
**DCM**	Dichloromethane
**TiPSOTf**	Triisopropylsilyl trifluoromethanesulfonate
**HOBt**	Hydroxybenzotriazole
**DIPEA**	*N*,*N*-Diisopropylethylamine
**HATU**	Hexafluorophosphate azabenzotriazoletetramethyl uronium
**DDQ**	2,3-Dichloro-5,6-dicyano-1,4-benzoquinone
**TrocCl**	2,2,2-Trichloroethoxycarbonyl chloride
**TFAA**	Trifluoroacetic anhydride
**MAO**	Monoamine oxidase
**SCR**	Succinate-cytochrome c reductase
**LPS**	Lipopolysaccharide
**IL**	Interleukin
**PTP**	Protein tyrosine phosphatase
**HSV-2**	*Herpes simplex* virus 2
**hBM-MSC**	Human bone marrow mesenchymal stem cells
**TFA**	Trifluoroacetic acid
**Et_3_N**	Triethylamine
**Et_2_O**	Diethyl ether
**NBS**	*N*-Bromosuccinimide
**AcOH**	Acetic acid
**Boc_2_O**	Di*-tert*-butyl dicarbonate
**Ac_2_O**	Acetic anhydride
**DMAP**	4-Dimethylaminopyridine
**TMSOTf**	Trimethylsilyl trifluoromethanesulfonate
**DMF**	Dimethylformamide
***n*-BuLi**	*n*-Butyl lithium
**EtOAc**	Ethyl acetate
***i*-BuOCOCl**	Isobutyl chloroformate
**DAST**	Diethylamino sulfur trifluoride
**DBU**	1,8-Diazabicyclo [5.4.0]undec-7-ene
**DIBAL-H**	Diisobutylaluminium hydride
**EDCl**	1-Ethyl-3-(3-dimethylaminopropyl)carbodiimide
**MsCl**	Methanesulfonyl chloride
**NCS**	*N*-Chlorosuccinimide
**TCDI**	Thiocarbonyldiimidazole
***m*-CPBA**	Meta-chloroperoxybenzoic acid
**AChE**	Acetylcholinesterase
**BChE**	Butyrylcholinesterase
**5-HT1A**	5-hydroxytryptamine 1A
**ZIKV**	Zika virus
**TCPTP**	T-cell protein tyrosine phosphatase
**RSV**	Respiratory syncytial virus
**ATP**	Adenosine triphosphate
